# Altered parabrachial nucleus nociceptive processing may underlie central pain in Parkinson’s disease

**DOI:** 10.1038/s41531-023-00516-x

**Published:** 2023-05-26

**Authors:** Arnaud Pautrat, Racha Al Tannir, Karin Pernet-Gallay, Rémi Soutrenon, Estelle Vendramini, Valérie Sinniger, Paul G. Overton, Olivier David, Véronique Coizet

**Affiliations:** 1 Université Grenoble Alpes, Inserm, U1216, CHU Grenoble Alpes, Grenoble Institut Neurosciences, 38000 Grenoble, France; 2 Department of Psychology, University of Sheffield, Sheffield, UK; 3 Aix-Marseille Université, Institut National de la Santé et de la Recherche Médicale, Institut de Neurosciences des Systèmes (INS) UMR1106, Marseille, 13005 France

**Keywords:** Parkinson's disease, Neurophysiology

## Abstract

The presence of central neuropathic pain in Parkinson’s disease suggests that the brain circuits that allow us to process pain could be dysfunctional in the disorder. However, there is to date no clear pathophysiological mechanism to explain these symptoms. In this work, we present evidence that the dysfunction of the subthalamic nucleus and/or substantia nigra pars reticulata may impact nociceptive processing in the parabrachial nucleus (PBN), a low level primary nociceptive structure in the brainstem, and induce a cellular and molecular neuro-adaptation in this structure. In rat models of Parkinson’s disease with a partial dopaminergic lesion in the substantia nigra compacta, we found that the substantia nigra reticulata showed enhanced nociceptive responses. Such responses were less impacted in the subthalamic nucleus. A total dopaminergic lesion produced an increase in the nociceptive responses as well as an increase of the firing rate in both structures. In the PBN, inhibited nociceptive responses and increased expression of GABA_A_ receptors were found following a total dopaminergic lesion. However, neuro-adaptations at the level of dendritic spine density and post-synaptic density were found in both dopaminergic lesion groups. These results suggest that the molecular changes within the PBN following a larger dopaminergic lesion, such as increased GABA_A_ expression, is a key mechanism to produce nociceptive processing impairment, whilst other changes may protect function after smaller dopaminergic lesions. We also propose that these neuro-adaptations follow increased inhibitory tone from the substantia nigra pars reticulata and may represent the mechanism generating central neuropathic pain in Parkinson’s disease.

## Introduction

Parkinson’s disease is characterized by a progressive degeneration of dopaminergic (DA) neurons located in the substantia nigra pars compacta (SNc), leading to the dysfunction of the basal ganglia. Parkinson’s disease is classically associated with motor symptoms, however it is increasingly recognized that non-motor symptoms, including pain, are very common. Pain is present in up to 85% of patients with Parkinson’s disease during the course of the disease^1,2^. Ford and collaborators^3^ distinguished between several pain types affecting groups of patients with Parkinson’s disease: musculoskeletal (~58.5%), radicular neuropathic (~38%), central neuropathic (~8.5–27%) pain, and pain induced by abnormal movements (dystonia, dyskinesia, ~33.5%). Pain threshold is also altered in patients with Parkinson’s disease, with or without pain symptoms^2,4–8^. Pain is negatively associated with quality of life in Parkinson’s disease^9^, with greater impact on quality of life than the motor impairment^1^. Pain is therefore an important concern in the management of the disorder.

Central neuropathic pain is described by patients as bizarre and unexplained painful sensations such as painful burning, stabbing, aching, itching or tingling sensations with no apparent origin, predominantly on the more affected side^10^. It is not directly related to the other pain symptoms described earlier^11^ that arise as a complication of the motor symptoms of the disorder. Central pain symptoms in Parkinson’s disease suggest that the brain circuits that allow us to perceive and process pain could be dysfunctional in Parkinson’s disease. While abnormal nociceptive processing has been reported in some cortical structures involved in pain^10,12^, there is to date no clear pathophysiological mechanism to explain central pain symptoms in Parkinson’s disease.

The parabrachial nucleus (PBN), a low level primary nociceptive structure in the brainstem, is a major central target for ascending nociceptive information from the spinal cord^13–15^. This structure has close connectivity with the basal ganglia^16–18^, yet it has been largely ignored in the context of Parkinson’s disease. PBN neurons project to one of the input structures of the basal ganglia, the subthalamic nucleus (STN)^16^, and receive a direct projection from the substantia nigra pars reticulata (SNr)^17^, a main output structure of the basal ganglia. Therefore, the PBN, STN and SNr are anatomically closely connected. We suggest that the dysfunction of these structures is important for central neuropathic pain in Parkinson’s disease. In particular, we suggest that the pathologically elevated activity in the STN in primate^19–21^ and rat models of Parkinson’s disease^16^ exacerbates the inhibitory tone coming from the SNr over its targets. Abnormal nociceptive processing has been shown in the STN of rodent models of Parkinson’s disease^16,22,23^ and nociceptive perception has recently been demonstrated to be modulated by optogenetic manipulation of the STN^23^. The dysfunction of the STN and SNr could therefore disrupt the ability of the PBN to process nociceptive inputs. Increased GABA release in the PBN from the SNr afferents is also likely to induce neuro-adaptations in the PBN as previously shown for another sensori-motor structure in the brainstem, the superior colliculus, which shares similar connectivity with the STN and SNr^24–26^. The PBN is a key nociceptive structure transmitting nociceptive information to higher brain areas involved in nociception such as the amygdala, the hypothalamus, the insular and cingulate cortices^27^. Therefore, dysfunction of the PBN is likely to have a substantial impact on the rest of the pain network and may underlie some of the unexplained painful sensations described by patients with Parkinson’s disease.

PBN involvement in nociception and the close connectivity of this structure with the basal ganglia is well established, however, demonstration of PBN dysfunction in the context of Parkinson’s disease remains to be determined. The main objective of the present work is to evaluate nociceptive processing in the PBN in a rodent model of Parkinson’s disease. Using rats with different degrees of dopaminergic (DA) cell loss in the SNc and denervation of the striatum, we measured PBN, STN and SNr nociceptive responses electrophysiologically. We then evaluated nociceptive perception in parkinsonian rats behaviorally. Finally, we completed the analysis by measuring markers of anatomical and neurochemical plasticity in the PBN that may explain our physiological results and may underlie the development of central neuropathic pain in Parkinson’s disease.

## Results

### Histology

Three experimental groups were defined according to the extent of the DA lesion induced by the toxin 6-hydroxydopamine (6-OHDA) in the SNc: Sham, Partial and Total DA lesions. The lesion was assessed by immunohistochemistry against tyrosine hydroxylase (TH) at both striatal and SNc levels. The sham group was composed of rats in which sterile saline had been injected unilaterally in the SNc instead of the toxin (Sham, *n* = 23). In the Partial DA lesion rats, TH-labeling was reduced to an average of 53.97 ± 4.06% (mean ± SEM) in the SNc and 68.06 ± 4.30% in the striatum (Partial DA lesion, *n* = 20), with the remaining DA neurons located in the medial part of the SNc. In the Total DA lesion group, only 10.52 ± 2.20% of TH-labeled neurons remained in the SNc on the lesioned side, leading to a remaining striatal innervation of 31.86 ± 4.40% (Total DA lesion, *n* = 22). The difference between these three groups in terms of percentage of remaining TH-labelling at both striatal and SNc levels was statistically significant. (Striatum: KW[2,62] = 49.47, *p* < 0.0001; SNc: KW[2,62] = 56.41, *p* < 0.0001). Dunn’s post-hoc test revealed that this difference was statistically significant between all pairs for both the striatum and the SNc (Sham vs Partial DA lesion, Sham vs Total DA lesion, Partial DA lesion vs Total DA lesion, Dunn’s *P* < 0.001) (Supplementary Fig. 1).

### Extracellular electrophysiology

We performed extracellular single unit recordings in the STN and PBN as well as extracellular multi-unit recordings in the SNr in the Sham, Partial and Total DA lesion groups to assess the impact of the DA lesions on their nociceptive responses and firing rates. The PBN recordings aimed at evaluating the functional state of this structure in the context of Parkinson’s disease, according to our main objective, while the STN and SNr recordings aimed at validating the abnormal functional states of these structures in our model as previously described in Parkinson’s disease patients and animal models. Similar to our previous protocols^16,18,24,26,28,29^, measurements were made during two phases of the stimulation trials: (1) During a control period, to evaluate the spontaneous firing rate of the recorded cells; (2) During a stimulation period, during which a noxious footshock was delivered at an intensity of 5.0 mA (0.5 Hz, 2 ms, 120 stimulations) to the contralateral back paw, to evaluate the nociceptive response of the recorded cells. The nociceptive nature of the stimulation has been demonstrated and fully described in our previous work^16,18,26^.

As illustrated in the Supplementary Fig. 2, we measured and compared the firing rate and various parameters of the phasic responses to the noxious stimulation (latency, duration, peak amplitude, magnitude) in the structures of interest in the three groups of animals (see method section for full details).

#### Nociceptive responses in the subthalamic nucleus – effect of the dopaminergic lesion

1. Recording sites and neurons - A Total of 61, 52 and 46 single cells were recorded across the STN in the Sham, Partial and Total DA lesion rats, respectively (Fig. 1a). The majority of the cells had an irregular firing pattern in the Sham and Partial DA lesion group while the Total DA lesion group had a higher percentage of cells with a bursting firing pattern (*p* < 0.05, *χ*² = 6.13) (Fig. 1b), a classical pathological electrophysiological marker for Parkinson’s disease^20^.

**Fig. 1:**
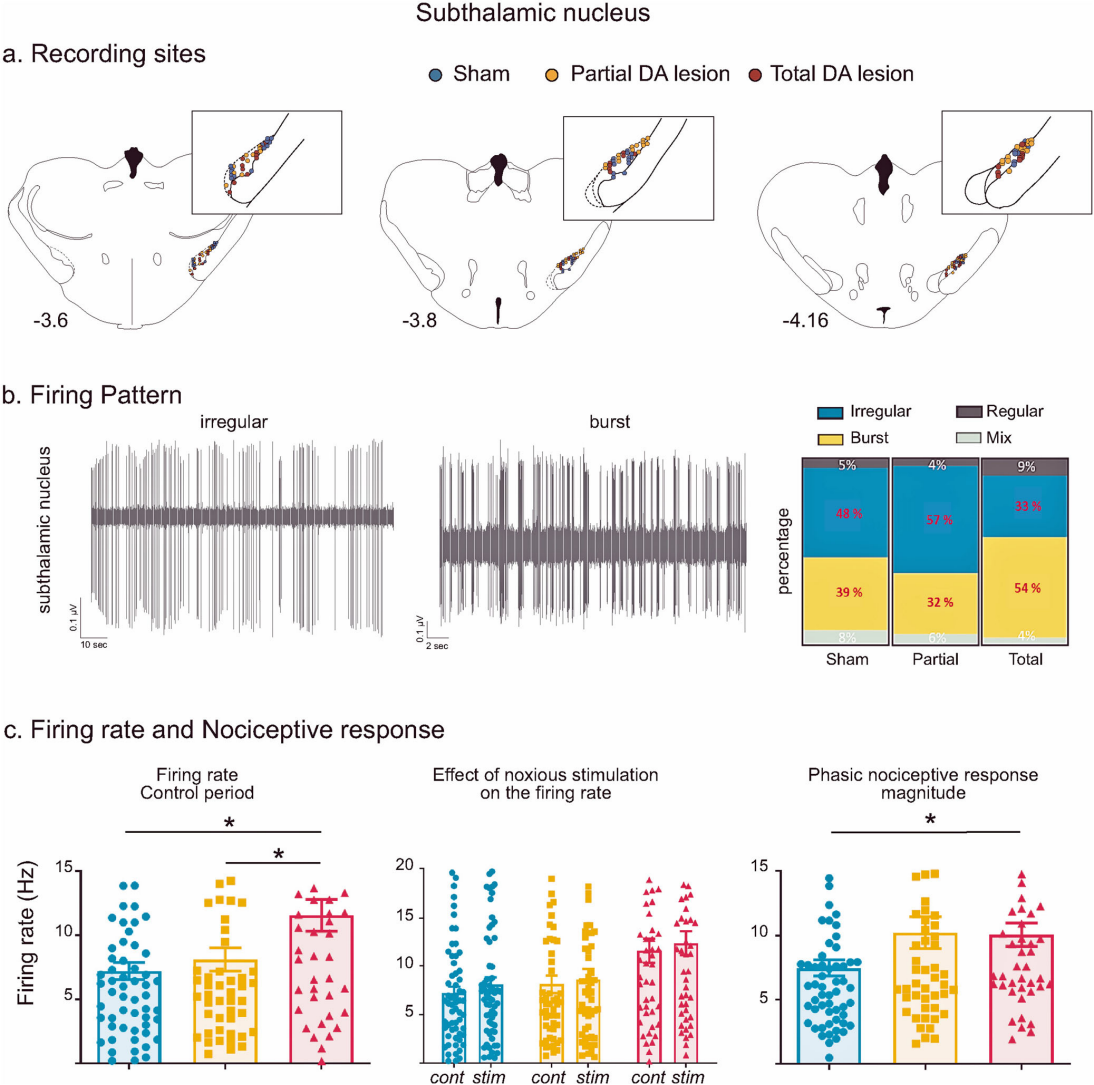
Subthalamic nucleus histological and electrophysiological results. **a** Location of recording sites within the STN for the Sham (blue dots), Partial DA lesion (orange dots) and Total DA lesion (red dots) groups. **b** *Left*: Individual recordings illustrating irregular and bursting firing patterns. *Right*: The proportion of irregular (blue), burst (yellow), regular (dark grey) and mixed (light grey) patterns recorded in the Sham, Partial and Total DA lesion groups (in percentage). Note that the Total DA lesion group is characterized by a higher number of bursting cells and lower number of irregular cells. **c** Histograms of the means (±SEM) of firing rate (*left*), STN firing rate measured during the control (cont) and noxious stimulation (stim) period (*middle*) and nociceptive response magnitude (*right*) measured in the Sham (blue), Partial (orange) and Total (red) DA lesion groups. Note that despite higher nociceptive responses in the Partial DA lesion group, this effect did not reach a statistical significance.
2. Firing rate—as illustrated in Fig. 1c, STN neurons recorded in the Total DA lesion group had a significantly higher spontaneous firing rate compared to the Sham or Partial DA lesion rats (mean ± SEM, Sham = 7.20 ± 0.67 Hz; Partial DA lesion = 8.10 ± 0.91 Hz; Total DA lesion = 11.48 ± 1.24 Hz; KW[2, 156] = 8.61, followed by Dunn’s post-hoc test, *p* < 0.05). The tonic nociceptive processing we have previously described in the STN was not disrupted by the DA lesion (See Supplementary Results I for details)^16^.
3. Phasic nociceptive responses—STN cells exhibited the same patterns of response to the footshock as previously described^16^. Responses to noxious stimulation in the STN universally began with an initial excitation and were predominantly short duration (mean ± SEM: 31.1 ± 1.8 ms) or long duration (mean ± SEM: 176 ± 20 ms) monophasic excitations at short latency (mean ± SEM: 26.2 ± 2.5 ms) (*n* = 106). The initial excitation could be followed by one (biphasic, *n* = 33) or two (triphasic, *n* = 20) additional inhibitory or excitatory phases (see Supplementary Results II and Supplementary Fig. 3 for full description and illustration of STN response types). The DA lesions led to a differential distribution of response types across the groups (*χ*² = 18.21, *P* < 0.05). The Partial DA lesion group had a higher number of biphasic responses, and both DA lesion groups had a higher number of long duration and lower number of short duration monophasic responses compared to the Sham animals. The nociceptive response magnitude was significantly increased in the Total DA lesion group compared to the Sham only (Sham = 7.48 ± 0.63 Hz; Partial DA lesion = 10.23 ± 1.24 Hz; Total DA lesion = 10.01 ± 0.92 Hz; KW[2, 156] = 6.32, followed by Dunn’s post-hoc test, *p* < 0.05) (Fig. 1c). Dopamine depletion did not alter other parameters of the phasic response.

##### STN results summary

Overall, STN cells in the Total DA lesion group showed an increased firing rate and nociceptive responses, as well as increased burst firing while the partial DA lesion group was not significantly impacted, despite the clear increase of the nociceptive response magnitude in this group (see Fig. 1c). The DA lesion changed the phasic nociceptive response types compared to the Sham rats with a reduction in monophasic short duration responses, an increase in monophasic long duration responses and additional phases in the response (such as biphasic excitations). The details of the firing rate and phasic response parameters (mean ± SEM) can be found in Supplementary Table 1.

#### Nociceptive responses in the substantia nigra pars reticulata—effect of the dopaminergic lesion

1. Recording sites—a Total of 124, 66 and 94 sites were recorded within the SNr in the Sham, Partial DA lesion and Total DA lesion animals respectively. The recording sites were mainly located centrally in SNr’s anterior-posterior axis (Fig. 2a).

**Fig. 2:**
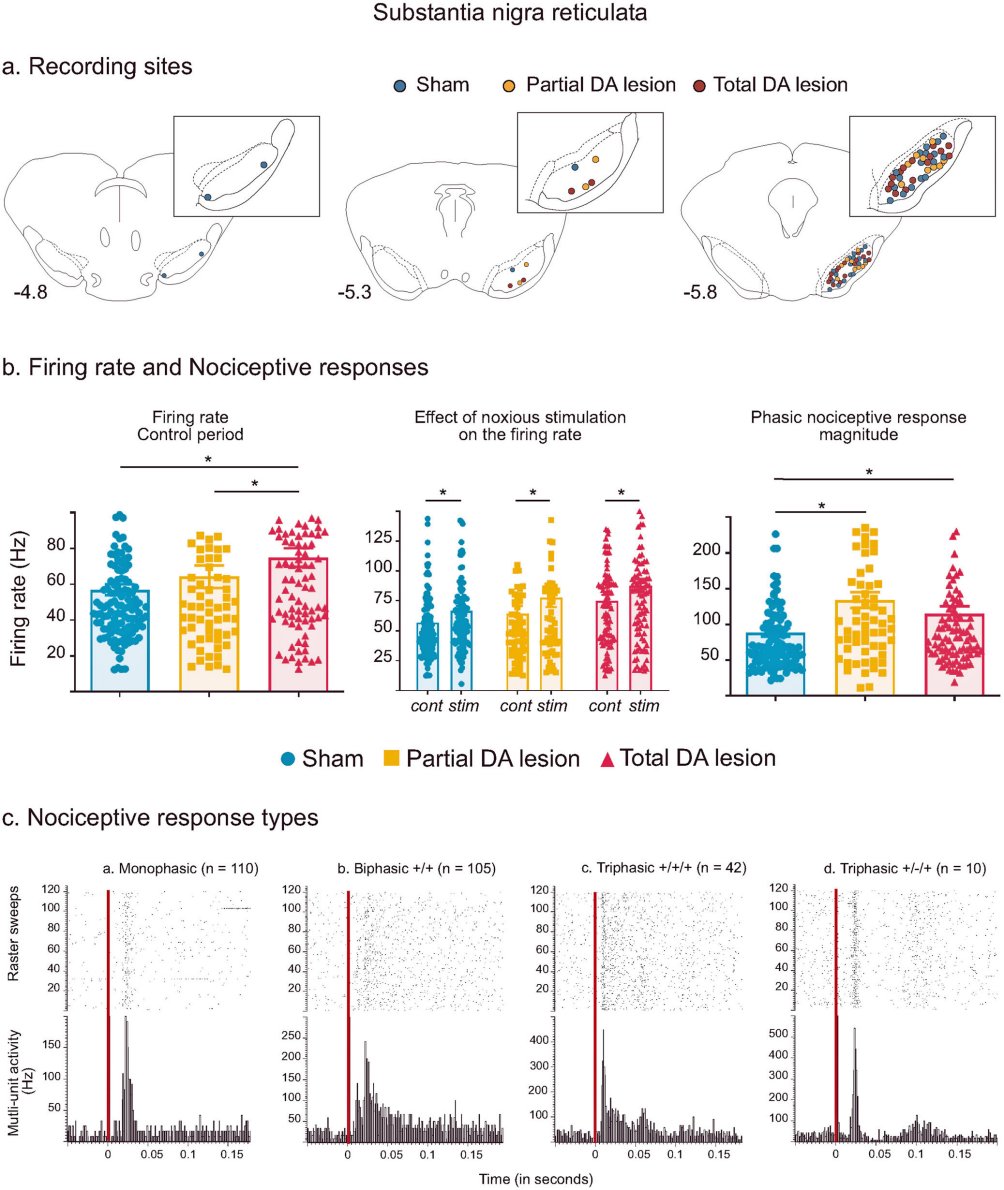
Subtstantia nigra pars reticulata histological and electrophysiological results. **a** Location of recording sites within the SNr for the Sham (blue dots), Partial DA lesion (orange dots) and Total DA lesion (red dots) groups. **b** C. Histograms of the means (±SEM) of firing rate (*left*), firing rate measured during the control (cont) and noxious stimulation period (stim) (*middle*) and nociceptive response magnitude (*right*) measured in the Sham (blue), Partial (orange) and Total (red) DA lesion groups. Note that the introduction of the noxious stimulation induced a firing rate increase in the three experimental groups. **c** Peristimulus histograms showing individual cases of different types of phasic noxious stimulation-evoked responses in the SNr. The red vertical line indicates the onset time of the noxious footshock. The *n* associated with each histogram indicates the number of cases exhibiting that class of response.
2. Firing rate—spontaneous multi-unit firing rate in the SNr was significantly increased in the Total DA lesion group when compared to the Sham and Partial DA lesion groups (mean ± SEM, Sham = 56.84 ± 3.03 Hz; Partial DA lesion = 63.60 ± 6.29 Hz; Total DA lesion = 74.93 ± 5.17 Hz; KW[2, 279] = 11.59, followed by Dunn’s post-hoc test, *p* < 0.01) (Fig. 2b). This parameter was not statistically different between the Sham and Partial DA lesion. The introduction of the noxious stimulation further increased SNr firing rate in all three experimental groups (Fig. 2b, Wilcoxon: *W* = −3562, *p* < 0.001) (See Supplementary Results III for details).
3. Phasic nociceptive responses—the noxious stimulation induced complex phasic responses in the SNr with several different patterns as illustrated in Fig. 2c. Responses to noxious stimulation began with an initial excitation and were predominantly short duration (mean ± SEM: 21 ± 0.001 ms) monophasic excitations at short latency (mean ± SEM: 14 ± 0.001 ms) (*n* = 119). The initial excitation could be followed by one (biphasic, *n* = 113) or two (triphasic, *n* = 52) additional inhibitory or excitatory phases (see Supplementary Results IV for a full description of response types). Phasic SNr responses to footshock were differentially distributed among the three experimental groups (*χ*² = 82.174, *p* < 0.001). Like the STN, both DA lesion groups had a smaller number of monophasic responses compared to Sham. The Partial DA lesion group had an increased number of excitatory biphasic responses and the Total DA lesion group had an increased number of excitatory triphasic responses.

SNr nociceptive responses were enhanced after the DA lesion. Analysis revealed a significant increase of nociceptive response magnitude (mean ± SEM, Sham = 88.72 ± 5.62 Hz; Partial DA lesion = 133.90 ± 10.92 Hz; Total DA lesion = 115.10 ± 9.86 Hz; KW[2, 279] = 16.71, followed by Dunn’s post-hoc test, *p* < 0.01) and peak amplitude (mean ± SEM, Sham = 286.90 ± 13.18 Hz; Partial DA lesion = 404.0 ± 28.58 Hz; Total DA lesion = 356.96 ± 21.75 Hz; KW[2, 279] = 16.53, followed by Dunn’s post-hoc test, *p* < 0.01) in both lesion groups compared to the Sham group. Nociceptive phasic responses recorded in the Total DA lesion group had significantly longer latencies compared to the Partial DA lesion rats (mean ± SEM, Sham = 13.11 ± 0.43 ms; Partial DA lesion = 12.64 ± 0.63 ms; Total DA lesion = 14.68 ± 0.49 ms; KW[2, 279] = 11.13, Dunn’s post-hoc test Partial DA lesion vs Total DA lesion: *p* < 0.01) while the duration of the response was not affected by the DA lesion.

##### SNr results summary

The DA lesions changed SNr responses to noxious stimuli, increasing the number of biphasic and triphasic patterns in the Partial and Total DA lesion groups, respectively. Furthermore, SNr nociceptive responses were increased in both DA lesion groups but the firing rate was only increased in the Total DA lesion group. The result suggests that the SNr output is enhanced in response to nociceptive input following a DA lesion in SNc, increasing the inhibitory tone that this structure will exert on its targets during noxious stimulation. The details of the firing rate and phasic response parameters (mean ± SEM) can be found in Supplementary Table 1.

#### Nociceptive responses in the parabrachial nucleus – effect of the dopaminergic lesion

1. Recording sites and neurons—a Total of 72, 73 and 49 single cells were recorded across the medial and lateral part of the PBN in Sham, Partial and Total DA lesion rats, respectively (Fig. 3a). The firing patterns of the majority of PBN neurons were either irregular or bursting (Fig. 3b). Unlike the STN, the proportions of irregular and bursting patterns in the PBN did not vary depending on the level of DA denervation (*χ*² = 4.11, p = 0.128) (See Supplementary Results V for details).

**Fig. 3:**
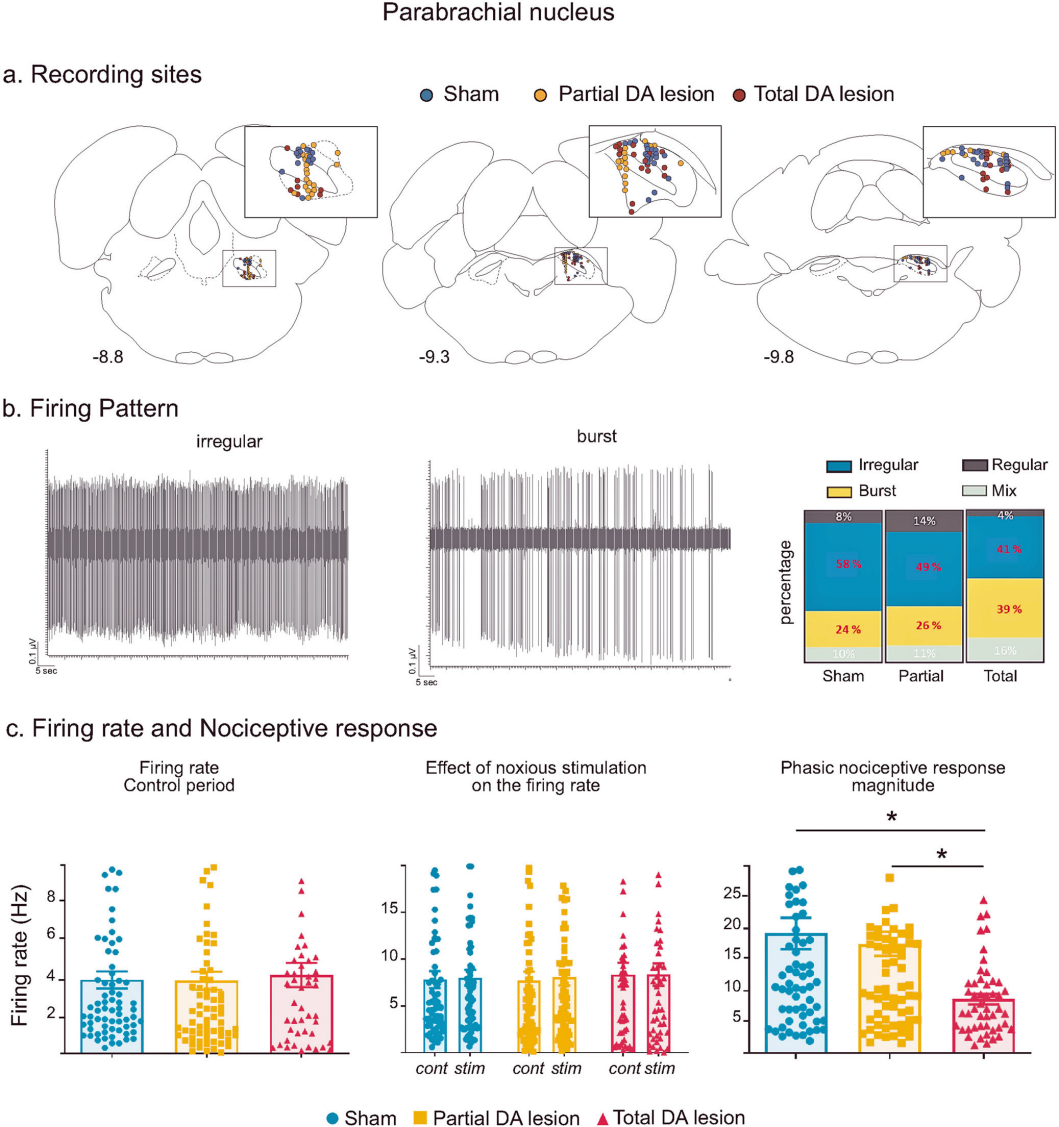
Parabrachial nucleus histological and electrophysiological results. **a** Location of recording sites within the PBN for the Sham (blue dots), Partial DA lesion (orange dots) and Total DA lesion (red dot) groups. **b** Left: Individual recordings illustrating irregular and bursting firing patterns. Right: The proportion of irregular (blue), bursting (yellow), regular (dark grey) and mixed (light grey) patterns recorded in the Sham, Partial and Total DA lesion rats. **c** Histograms of the means (±SEM) of firing rate (*left*), firing rate during the control (cont) or stimulation (stim) period (*middle*) and nociceptive response magnitude (*right*) measured in the Sham (blue), Partial (orange) and Total (red) DA lesion groups.
2. Firing rate—the firing rate of PBN cells was not altered in DA depleted animals when compared with Sham animals (mean ± SEM, Sham = 7.81 ± 0.91 Hz; Partial DA lesion = 7.71 ± 0.96 Hz; Total DA lesion = 8.32 ± 1.28 Hz, KW[2, 191] = 0.75) (Fig. 3a). In addition, noxious stimulation did not change the firing rate of PBN neurons in the three experimental groups (mean ± SEM, Sham: no stimulation = 7.81 ± 0.91 Hz vs stimulation 8.01 ± 0.85 Hz; Partial DA lesion: no stimulation: 7.71 ± 0.96 Hz vs stimulation 8.03 ± 0.85 Hz; Total DA lesion: no stimulation: 8.32 ± 1,28 Hz vs stimulation 8.34 ± 1.20 Hz, 2-way ANOVA F[1, 382] = 0.050, *p* = 0.82).
3. Phasic nociceptive responses—following noxious stimulation, PBN neurons showed various complex phasic responses which, as with the STN and the SNr, also began with an initial excitation. Responses to noxious stimulation in the PBN were predominantly short duration (mean ± SEM: 28.4 ± 0.004 ms) monophasic excitations at short latency (mean ± SEM: 18.69 ± 0.17 ms) (*n* = 88). The initial excitation could be followed by one (biphasic, *n* = 39) or two (triphasic, *n* = 17) additional inhibitory or excitatory phases (see Supplementary Results VI and Supplementary Fig. 4 for full description and illustration of response types). PBN phasic response types were not differently distributed among the three experimental groups (*χ*² = 6.52, p = 0.163). However, analysis revealed significantly longer nociceptive response latencies in both DA lesion groups compared to the Sham animals (mean ± SEM, Sham = 11.97 ± 1.15 ms; Partial DA lesion = 16.53 ± 1.05 ms; Total DA lesion = 26.64 ± 3.15 ms, KW[2, 191] = 33.98, followed by Dunn’s post-hoc test, *p* < 0.01). As to the nociceptive responses themselves, the DA lesion affected the responses in the Total DA lesion group only, compared to the Sham and Partial DA lesion groups, with a significant reduction of the magnitude (mean ± SEM, Sham = 19.19 ± 2.53 Hz; Partial DA lesion = 17.53 ± 1.94 Hz; Total DA lesion = 8.67 ± 0.83 Hz, KW[2, 191] = 13.59, followed by Dunn’s post-hoc test, *p* < 0.01) and peak amplitude (mean ± SEM, Sham = 58.33 ± 7.87 Hz; Partial DA lesion = 44.59 ± 4.18 Hz; Total DA lesion = 27.55 ± 2.65 Hz; KW[2, 191] = 10.05, followed by Dunn’s post-hoc test, *p* < 0.01). The nociceptive response parameters of the Partial DA lesion animals were not significantly different to those of the Sham animals (Fig. 3c).

##### PBN results summary

Overall, the DA lesion did not alter PBN firing rate. PBN nociceptive response latencies were longer in both DA lesion groups, the nociceptive response magnitude and peak amplitude were reduced in the Total DA lesion group only. The details of the firing rate and phasic response parameters (mean ± SEM) can be found in Supplementary Table 1.

#### Nociceptive signal transmission

Our hypothesis was that nociceptive information coming from the spinal cord would first be processed by the PBN, then transmitted to the STN and finally to the SNr. However, analysis of the response latencies did not support this organization. In the Sham rats, the PBN and SNr nociceptive response latencies were both significantly shorter than those of the STN (Mean ± SEM: STN = 33.28 ± 4.97 ms, SNr = 13.11 ± 4.29 ms, PBN = 11.97 ± 1.15 ms; Kruskal Wallis: KW[2, 254] = 36.79, followed by Dunn’s post-hoc test, *p* < 0.001). Nociceptive response latencies were also significantly shorter in the PBN than in the SNr (Dunn’s post-hoc test, *p* < 0.01). These relationships were also found within the Partial and Total DA lesion groups (Partial DA lesion: KW [2, 189] = 36.42, *p* < 0.001; Total DA lesion: KW [2, 186] = 7.15, followed by Dunn’s post-hoc test, *p* < 0.05). The result suggests that the SNr is processing nociceptive signals, possibly coming from the PBN, before the STN and may thus represent another input nucleus of the basal ganglia at least for this type of aversive information.

#### Cross-correlation analysis

Implanting electrodes in each structure in the same rat allowed us to simultaneously record the neuronal activity of at least two structures and to perform cross-correlation analysis to explore their connectivity. Abnormally synchronized activity at multiple levels of the basal ganglia–cortical loop architecture is a hallmark of Parkinson’s disease and this excessive synchronization correlates with the extent of the motor deficit^30^. As a secondary objective, we therefore tested whether abnormal similar synchronized activity can be found between the PBN and the STN, the STN and the SNr and between the SNr and the PBN in the lesioned animals. To do so, we constructed cross-correlation histograms of activity in pairs of structures in the three experimental groups. The presence of peaks of excitation is a marker of correlated activity. A summary of the method and examples of cross-correlation histograms are illustrated in the Supplementary Fig. 5.

- Cross-correlation analysis during the control period:

Contingency analysis on cross-correlation histograms between the STN-PBN, STN-SNr and PBN-SNr in the sham group revealed a significant difference (*χ*² = 14.09, *p* < 0.001) due to a higher number of pairs with cross-correlated activity between the STN and the SNr (STN-SNr: 42%, 14/33; STN-PBN: 8%, 4/48); (PBN-SNr: 12%, 4/33). The number of cross-correlated pairs between the PBN-SNr was significantly higher for both DA lesion (Sham: 12%, 4/33; Partial DA lesion: 30%, 19/63; Total DA lesion: 49%, 22/45; *χ*² = 12.01, *p* < 0.01) but not between the STN-PBN (Sham: 8%, 4/48; Partial DA lesion: 19%, 5/27; Total DA lesion: 5%, 1/21), or between the STN-SNr (Sham: 42%, 14/33; Partial DA lesion: 45%, 9/20; Total DA lesion: 24%, 8/34).

- Cross-correlation analysis during the stimulation period:

Cross-correlated activity significantly increased in the sham group for all pairs of structures during a nociceptive stimulation (control vs stimulation periods: STN-PBN: 8%, 4/48 vs 42%, 20/48, *χ*² = 14.22, *p* < 0.001; STN-SNr: 42%, 14/33 vs 70%, 23/33, *χ*² = 4.98, *p* < 0.05; PBN-SNr: 12%, 4/33 vs 58%; 19/33, *χ*² = 12.01, *p* < 0.01). This increase was not found in the Partial and Total DA lesion groups when analyzing the STN-PBN cross-correlations (control vs stimulation periods: Partial DA lesion group: 19%, 5/27 vs 19% 5/26; Total DA lesion group: 5%, 1/21 vs 0%, 0/21), but was significant in the Total DA lesion group (but not the Partial lesion group) for STN-SNr pairs (control vs stimulation periods: Total group, STN-SNr: 24%, 8/34 vs 79%, 27/34, *χ*² = 21.25, *p* < 0.001; Partial group, STN-SNr: 45%, 9/20 vs 55%, 11/20, *p* > 0,05) and was significant for the partial DA lesion group (but not the Total DA lesion group) for the PBN-SNr pairs (control vs stimulation periods: Partial, PBN-SNr: 30%, 19/63 vs 67%, 42/63, *χ*² = 3.87, *p* < 0.05; Total, 49%, 22/45 vs 60%, 27/45, *p* > 0,05).

##### Cross-correlation analysis summary

Overall, in the Sham group, cross-correlated activity was found between the STN and the SNr during the control period. The DA lesion does not alter this STN-SNr pattern but induced an increase in abnormal correlated activity specifically between the PBN and the SNr. Nociceptive stimulation generally increased the occurrence of correlated firing between all three structures in the Sham group and the DA lesion disrupted this aspect of neuronal processing. The details of the percentage of correlated and un-correlated activity between each pair of structure for the three experimental groups can be found in Supplementary Table 2.

### Electrophysiology intermediate conclusion

The electrophysiological results revealed that a DA lesion in the SNc altered neuronal activity in the STN after a total DA lesion, while activity in the SNr was impacted in both the Partial and Total DA lesion group. SNr phasic nociceptive responses were enhanced in both groups, while a total DA lesion gave rise to the classical increased firing rate in this structure. PBN neurons showed decreased phasic nociceptive responses in the Total lesion group, which is consistent with the general tonic and phasic hyperactivity in the SNr, sending inhibitory projections to the PBN. However, rats in the Partial DA lesion group had normal nociceptive responses in the PBN, albeit with slightly longer latencies, despite increased nociceptive activity in the SNr.

These results added two additional questions to our primary objective:

- What is the effect of DA lesions in SNc on nociceptive perception?

PBN nociceptive responses were inhibited in the Total DA lesion group but the Partial DA lesion group had similar nociceptive responses to the Sham group indicating a possible nociceptive impairment in the Total DA lesion group. We thus tested whether the DA lesion impacted the nociceptive perception behaviorally, expecting the Total DA lesion group to be impaired, but not the Partial DA lesion group.

- Does a DA lesion in the SNc induce neuro-adaptive plasticity in the PBN?

The lack of effect on PBN activity in the Partial DA lesion group, previously described, could be explained by insufficient abnormal inhibitory tone coming from the SNr in response to the DA lesion, leaving PBN activity unaffected. However, we have previously shown that another sensory-motor structure in the brainstem, the superior colliculus, sharing similar anatomical connections with the basal ganglia^24,25,31^, shows neuroplasticity in the 6-OHDA rat model of Parkinson’s disease^26^. Therefore, a similar neuro-adaptation may have also occurred in the PBN, allowing this structure to process nociceptive information normally in the rats in the Partial DA lesion group. We thus used different physiological, anatomical and molecular approaches to test whether a neuro-adaptation had taken place in the PBN in DA lesioned animals.

Both questions have been addressed in the following analysis.

### Behavioral evaluation

**Fig. 4:**
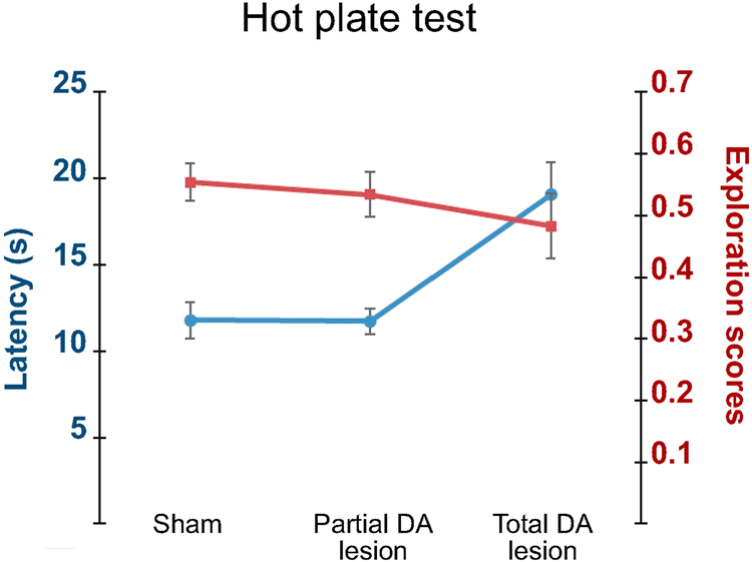
Effect of dopamine depletion in the hot plate test. Mean latencies (±SEM—blue curves) to produce the first sign of paw licking and foot stomping (the most frequent responses) to the heat of the plate (53°) and mean exploratory scores (±SEM—red curves) measured in the Sham (*n* = 14), Partial (*n* = 13) and Total (*n* = 12) DA lesion groups.

To evaluate the effect of SNc DA lesions on nociceptive processing behaviorally, we tested the Sham (*n* = 14), Partial (*n* = 13) and Total (*n* = 12) DA lesion rats behaviorally using the hot-plate test. The Total DA lesion group showed significantly longer latencies to produce the first sign of paw licking and foot stomping (the most frequent responses), jumping or vocalization, when compared to the Sham or rat in the Partial DA lesion group (mean ± SEM, Sham = 11.79 ± 1.03 s; Partial DA lesion = 11.73 ± 0.75 s; Total DA lesion = 19.07 ± 1.87 s; KW[2,35] = 13.00, followed by Dunn’s post-hoc test, *p* < 0.01) (Fig. 4).

We compared the exploratory behavior (i.e. rearing, full turn) between the three groups of rats during the hot plate test to check whether the longer latencies described before could be due to a motor impairment. We did not find a significant difference between the Sham, Partial and Total DA lesion rats in their exploratory scores (KW[2,35] = 5.02; *p* = 0.082) (Fig. 4).

### Electrophysiological, neuroanatomical and molecular evaluation of PBN neuroplasticity

#### Extracellular electrophysiology

We first tested the presence of a nociceptive sensory rebound^26^ in the PBN, in this case defined as an enhanced sensory responsiveness following suppression of the inhibitory influence of the SNr. In our experiment, the activity of the SNr was reduced with a local injection of muscimol, a GABA_A_ agonist (withdrawal). The sudden reduction of SNr hyperactivity should reveal neuroplasticity in the PBN in the form of increased nociceptive responses (sensory rebound) in lesioned animals relative to Sham.

Simultaneous recordings were made from the SNr (multi-unit activity) and PBN (single unit) neurons of Sham (*n* = 8), Partial (*n* = 7) and Total (*n* = 8) DA lesion rats.

**Fig. 5:**
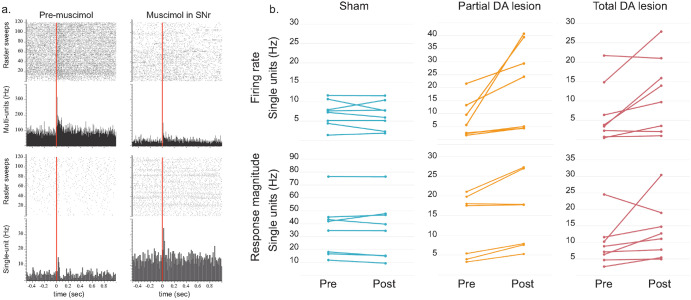
Sensory rebound – Effect of muscimol injection in the substantia nigra pars reticulata on the parabrachial nucleus firing rate and nociceptive responses. **a** Peri-stimulus histograms and raster sweeps showing individual examples of nociceptive responses recorded in a Total DA lesion rat simultaneously in the SNr (*top*) and PBN (*bottom*), before (*left*) and after (*right*) muscimol injection in the SNr. The red line represents the onset of the noxious footshock. **b** Individual plots of the mean (±SEM, Hz) firing rate (*top*) and nociceptive response magnitude (*bottom*) before (pre) and after (post) muscimol injection in the SNr measured in the Sham (blue), Partial (orange) and Total (red) DA lesion groups. Note that this injection has no significant effect on PBN activity in the Sham group while both DA lesion groups presented a strong sensory and firing rate rebound.

- Effect of intra-reticulata muscimol on SNr activity—injections of muscimol into the SNr significantly reduced the local firing rate in the Sham, Partial and Total DA lesion groups (Mean ± SEM: Sham pre = 55.15 ± 6.98 Hz vs post = 42.67 ± 9.46 Hz, *p* < 0.05; Partial pre = 38.87 ± 2.37 Hz vs post = 25.66 ± 5.15 Hz, *p* < 0.05; Total: pre = 68.39 ± 12.87 Hz vs post = 29.05 ± 9.32, *p* < 0.001, two-way ANOVA F[1,20] = 30,83 followed by Bonferroni’s correction for multiple comparisons). It also reduced the nociceptive responses in the three experimental groups with a significant decrease in the magnitude (Mean ± SEM: Sham pre = 96.59 ± 13.67 Hz vs post = 60.59 ± 15.62 Hz, *p* < 0.01; Partial pre = 34.76 ± 1.07 Hz vs post = 14.61 ± 1.00 Hz, *p* < 0.05; Total pre = 127.17 ± 14.12 Hz vs post = 65.46 ± 9.6 Hz, *p* < 0.05, two-way ANOVA on repeated measures F[1,20] = 36,56, followed by Bonferroni’s correction for multiple comparisons) and peak amplitude (Mean ± SEM: Sham pre = 254.54 ± 21.22 Hz vs post = 158.88 ± 21.36 Hz, *p* < 0.01; Partial pre = 153.57 ± 5.41 Hz vs post = 90.48 ± 17.58 Hz, *p* < 0.05; Total pre = 308.27 ± 34.61 Hz vs post = 146.19 ± 22.75 Hz, *p* < 0.01, two-way ANOVA on repeated measures F[1,20] = 46,39, followed by Bonferroni’s correction for multiple comparisons).
- Effect of intra-reticulata muscimol on PBN activity - The depression of SNr neuronal activity and nociceptive responses by muscimol had differential effects on the activity of PBN neurons depending on the experimental group tested. PBN firing rate and nociceptive responses were not changed following SNr blockade in the Sham group (Mean ± SEM: firing rate pre = 7.02 ± 1.18 Hz vs post = 6.61 ± 1.23 Hz, *p* > 0.99, magnitude pre = 36.05 ± 7.42 Hz vs post = 35.66 ± 7.88 Hz, *p* > 0.99, two-way ANOVA on repeated measures F[1,20] = 11,38, followed by Bonferroni’s correction for multiple comparisons; the other parameters of the response were also not significantly affected). In contrast, as illustrated in Fig. 5, both the Partial and Total DA lesion groups showed increased PBN firing rates after the injection of muscimol in the SNr (Mean ± SEM: Partial pre = 8.08 ± 2.77 Hz vs post = 21.09 ± 6.20 Hz, *p* < 0.05; Total pre = 6.73 ± 2.68 Hz vs post = 11.66 ± 3.37 Hz; *p* < 0.05, two-way ANOVA on repeated measures F[1,20] = 11,38, followed by Bonferroni’s correction for multiple comparisons) in addition to increased nociceptive response magnitudes (Mean ± SEM: Partial pre = 12.73 ± 3.05 Hz vs post = 15.80 ± 3.46 Hz, *p* < 0.05; Total pre = 8.99 ± 2.42 Hz vs post = 11.05 ± 1.70 Hz, *p* < 0.01, two-way ANOVA on repeated measures F[1,20] = 12,13, followed by Bonferroni’s correction for multiple comparisons). The maximum amplitude was increased significantly in the Total DA lesion group (Mean ± SEM: pre = 23.52 ± 7.07 Hz vs post = 32.83 ± 6.2 Hz, W = −22, *p* < 0.05), and there was a trend toward an increase in the Partial DA lesion group (Mean ± SEM: pre = 37.62 ± 10.02 Hz vs post = 45.00 ± 12.57 Hz W = −13, *p* = 0.062).

#### Golgi-Cox staining

**Fig. 6:**
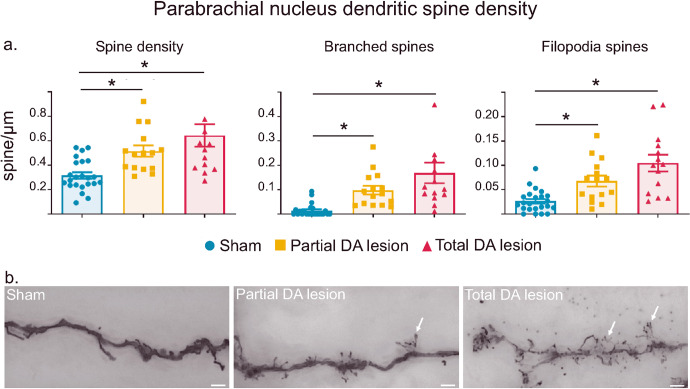
Parabrachial nucleus spine density and morphology. **a** Histograms of the means (±SEM) of spine density (*left*), branched spine density (*middle*) and filopodial spine density (*right*) measured using Golgi-Cox in the Sham (blue), Partial (orange) and Total (red) DA lesion groups. **b** Photomicrographs of dendritic spines labelled with Golgi-Cox staining in the Sham (*left*), Partial (*middle*) and Total (*right*) DA lesion groups. Note the presence of numerous branched spines in both DA lesion groups (white arrows). Scale bar = 10 μm; magnification 400×.

The nociceptive sensory rebound in the PBN in DA lesioned animals following suppression of SNr activity suggests that neuro-adaptations had occurred in the PBN following the DA lesion. To assess that possibility, we first focused on dendritic spines, a well-recognized locus of change in neuroplasticity. We counted, measured and categorized spines according to their morphology based on their length/width ratio as described by Risher and collaborators^32^. An average of ~600 µm of dendrite from Sham (*n* = 5), Partial DA lesion (*n* = 4) and Total DA lesion (*n* = 3) rats were analyzed. Analysis revealed that both DA lesion groups showed a significant increase of the spine density (spines/µm: Mean ± SEM Sham = 0.32 ± 0.03; Partial = 0.52 ± 0.05; Total = 0.64 ± 0.09, KW[2,49] = 18.06, followed by Dunn’s post-hoc test, *p* < 0.0001), at least partially accounted for by an increase in branched spine density (spine/µm: Mean ± SEM Sham = 0.014 ± 0.0054; Partial = 0.098 ± 0.018; Total = 0.17 ± 0.042; KW[2,49] = 30.94, followed by Dunn’s post-hoc test *p* < 0.0001), and filopodia spine density (spines/µm: Mean ± SEM Sham = 0.026 ± 0.0048; Partial = 0.068 ± 0.011; Total lesion = 0.10 ± 0.017; KW[2,49] = 20.17, followed by Dunn’s post-hoc test, *p* < 0.0001) when compared to the Sham animals (Fig. 6).

The density of the stubby spines (spines/µm: Mean ± SEM Sham = 0.060 ± 0.0092; Partial = 0.072 ± 0.011; Total = 0.058 ± 0.015; KW [2,49] = 1.98, *p* = 0.37), and thin spines density (spines/µm: Mean ± SEM Sham = 0.074 ± 0.0095; Partial = 0.072 ± 0.011; Total = 0.058 ± 0.015; KW[2,49] = 3.41, *p* = 0.18) were not significantly altered in both DA lesion groups when compared to the Sham group.

#### Electron microscopy

Our electrophysiological experiments showed that a DA lesion in the SNc increased SNr firing rate in animals in the Total DA lesion group and enhanced SNr nociceptive responses in animals in both the Partial and Total DA lesion groups. SNr projections to its targets have been shown to be mainly of GABAergic nature^33^. We therefore tested if increased activity in the SNr induced a modification in the postsynaptic densities (PSD) in the PBN, given that morphological changes in the PSD, particularly where parameters are reduced, are associated with an increase in inhibitory communication. The scaffolding proteins at the synaptic complex are specific to the type of receptors expressed in that area. PSD-95 and gephyrin scaffolding proteins anchor glutamatergic and GABAergic receptors to the postsynaptic membrane, respectively. Gephyrin and PSD-95 act in opposite directions^34^. In the presence of increased GABA receptor recruitment, more gephyrin is expressed at the synaptic complex which reduces the cluster of PSD-95 and in turn the size of the PSD^34^.

**Fig. 7:**
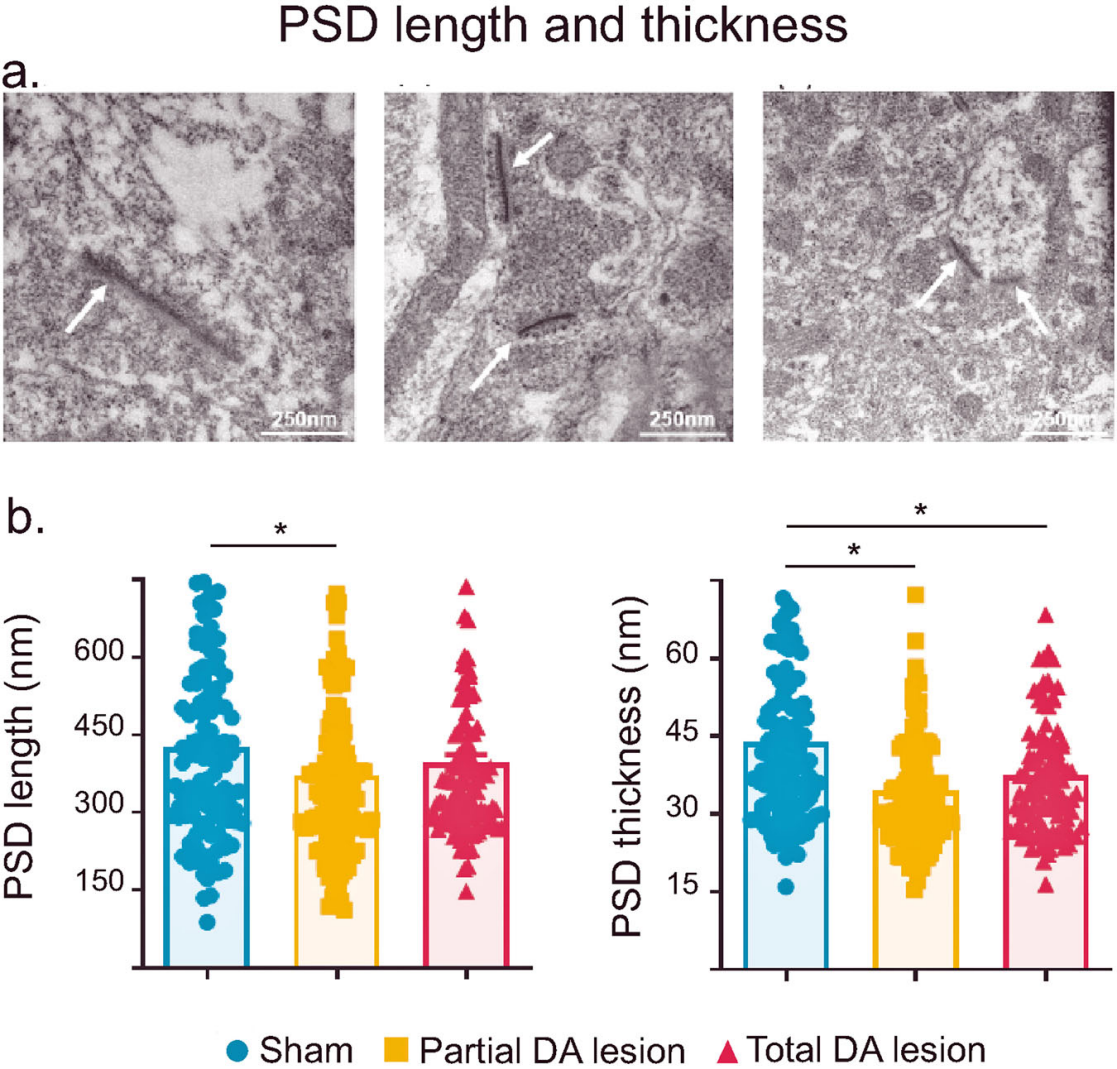
Parabrachial nucleus post synaptic density. **a** Representative photomicrographs of post-synaptic densities in Sham (*left*), Partial (*middle*) and Total (*right*) DA lesion rats indicated by the white arrows. Note that the lengths of the PSD are clearly shorter in DA lesioned animals compared to the shams. **b** Histograms of the mean (±SEM) PSD length (*left*) and thickness (*right*) measured using electron microscopy in the Sham (blue), Partial (orange) and Total (red) DA lesion groups.

In each experimental group, we analyzed synaptic morphology at the ultrastructural level. We measured 50 PSD lengths and widths per animal (three animals per group), mainly located on the shaft of the dendrite. Statistical analysis comparing Sham and DA lesioned rats showed a main effect of the lesion on PSD thickness (Mean ± SEM Sham = 43.84 ± 1.49 nm; Partial = 34.53 ± 1.13 nm; Total = 37.49 ± 1.25 nm; KW[2, 357] = 28.18; *p* < 0.0001) and a strong trend toward an effect on PSD length (Mean ± SEM Sham = 427.30 ± 17.75 nm; Partial = 372.20 ± 14.52 nm; Total = 396.10 ± 15.84 nm; KW[2, 357] = 5.73; *p* = 0.05). Dunn’s multiple post-hoc comparisons revealed a significant reduction in PSD thickness in the Partial DA lesion (*p* < 0.0001) and Total DA lesion group (*p* < 0.01), and a close to significant reduction in PSD length the Partial DA lesion group (*p* = 0.05) when compared to the Sham group (Fig. 7).

#### Western Blot

**Fig. 8:**
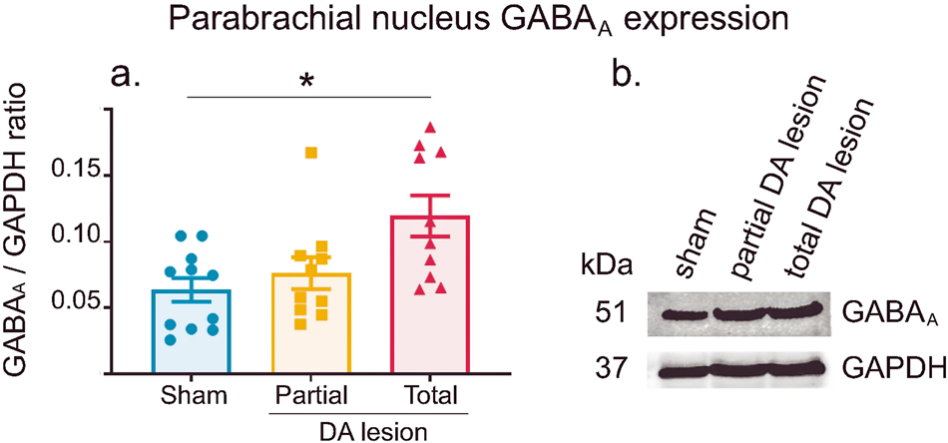
Parabrachial nucleus GABA_A_ receptor expression. **a** Histograms of the mean (±SEM) of GABA_A_/GAPDH ratio measured using Western Blot in the Sham (blue), Partial (orange) and Total (red) DA lesion groups (left). **b** Individual examples of Western Blot immuno-expression against GABA_A_ protein and GAPDH as a protein migration control (right).

DA lesions increased SNr firing rate in the Total DA lesion animals and exacerbated SNr nociceptive responses in both Partial and Total DA lesion groups. Given that SNr projections to its targets are mainly of GABAergic nature^33^, we tested if the increased activity in the SNr following the DA lesions induced a modification in the expression of GABAergic receptors in the PBN. The GABA_A_ receptor is the most widespread receptor at inhibitory synapses such as the ones present in the PBN and the level of GABA_A_ receptors at inhibitory synapses fluctuates based on the level of synaptic activity—the more active the synapse, the higher the level of GABA_A_ receptors within that synapse^35^. Western blot analysis was therefore used to detect and compare the possible changes in GABA_A_ receptors in the PBN of the 3 groups. The GABA_A_ receptor antibody we used recognizes GABA_A_ receptors in rats, mice and humans. This polyclonal antibody was raised against amino acids 1–15 of mature rat GABA_A_ receptor α1 subunit containing a cysteine at the C-terminus for linkage to a carrier protein. The predicted molecular weight for the rat GABA_A_ α1 subunit is 51 kDa, and it forms part of the pentameric structure in the full receptor. GAPDH protein (37 kDa) served as a housekeeping protein and was used for the Integrated Optical Density (IOD) analysis of GABA_A_. For this experiment, we compared the Sham (*n* = 11), Partial (*n* = 10) and Total (*n* = 10) DA lesion groups and found a significant difference in the expression of PBN GABA_A_ receptors between the three experimental groups (Mean ± SEM Sham = 0.064 ± 0.009; Partial = 0.076 ± 0.012; Total = 0.119 ± 0.015; ANOVA, F[2:28] = 5.71, *p* < 0.01). This effect was due to a significantly higher expression of GABA_A_ receptors in the Total DA lesion group compared to the Sham group (Tukey’s post-hoc test, *p* < 0.05), but not when compared to the Partial DA group. The Sham and Partial DA lesion groups were not statistically different (Fig. 8).

**Fig. 9:**
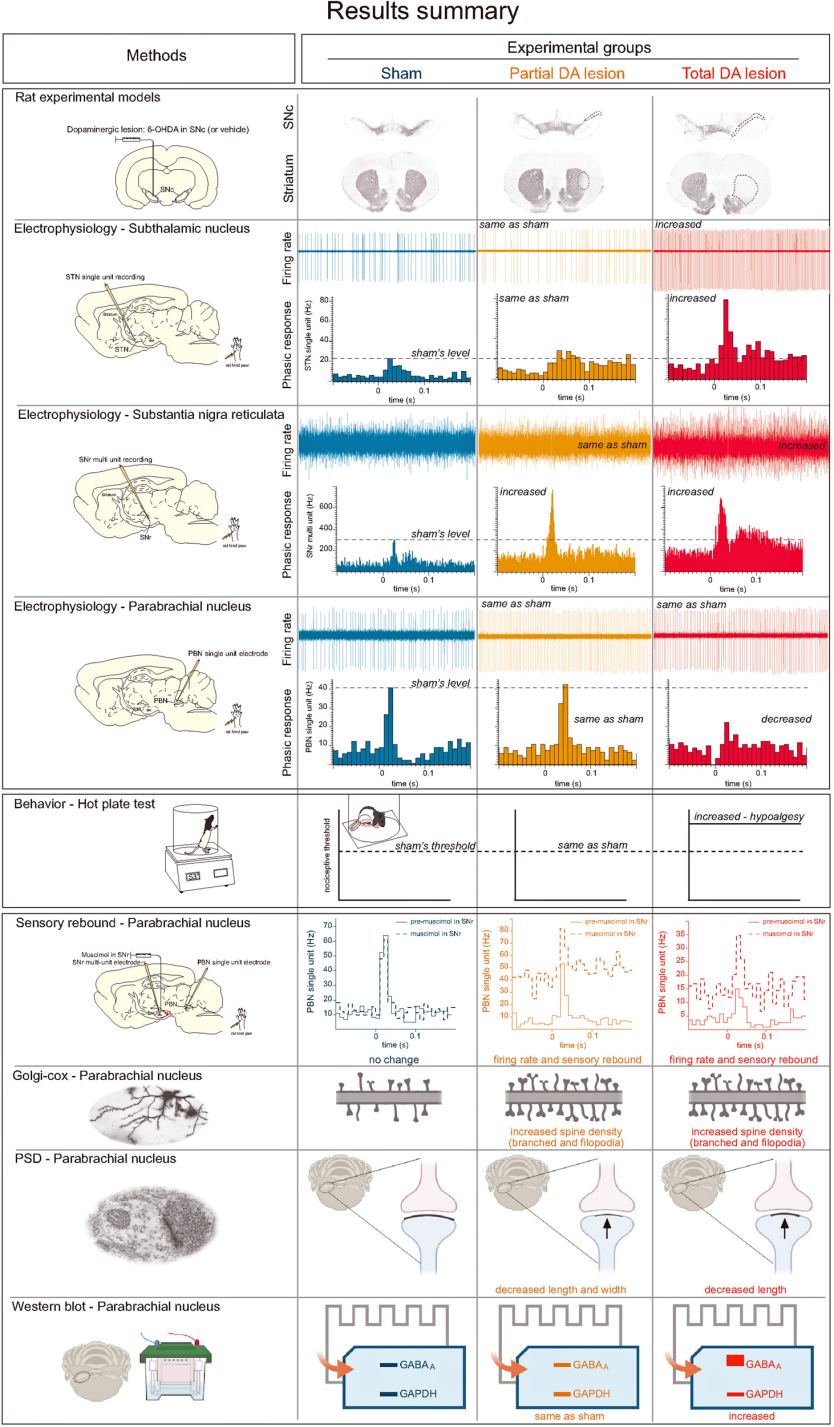
Summary of the main results. Schematic illustration of the main results according to the methods and experimental groups.

The main results of the present work are summarized in the Fig. 9.

## Discussion

Based on our earlier data^16^ and evidence from the literature^17,36^, we previously described the presence of a novel nociceptive network closely linking the PBN, the STN and the SNr^16^. We suggested that this network may be important in the development of central neuropathic pain observed in Parkinson’s disease. However, a main issue was that - unlike the STN - nociceptive activity in the PBN and SNr had never been characterized in the context of Parkinson’s disease. The demonstration of a dysfunction in each element of this network was a crucial first step in support of the contention that the network is involved in pain symptoms in Parkinson’s disease. The present work has led to several important and previously unreported fundamental pre-clinical findings which, combined together, allowed the elaboration of new mechanistic hypothesis not only for central pain symptoms, but also for pain sensitivity at a clinical level.

Our studies were based on the postulate that a dysfunction of the basal ganglia may have an impact on the nociceptive processing in the PBN, following the classical pathophysiological changes underlying Parkinson’s disease^19–21,37^. We thus recorded the neuronal alterations in the SNr and the STN following different degrees of DA denervation, to ascertain and characterize the dysfunction of these structures in our models. We found that partial or total DA lesions in the SNc did not have the same effect on STN and SNr neuronal activity. Partial DA loss increased the phasic nociceptive responses in the SNr, while STN nociceptive responses were less impacted (not statistically different from the Sham group and from the Total DA lesion group). Total DA loss extending over the SNc enhanced both phasic nociceptive responses and firing rate in both the STN and the SNr. Hence, our results demonstrate that these two key structures of the basal ganglia indeed have exacerbated neuronal activity in the context of Parkinson’s disease. However, our models also show that the SNr is dysfunctional before the STN, since a small DA lesion had no clear effect on the STN.

In Parkinson’s disease, a key pathophysiological change is that the DA degeneration leads to an increased firing rate in STN glutamatergic neurons which consequently increases the firing rate of SNr GABAergic neurons via the well-known connectivity between these structures. However, the literature also indicates that early dysfunction of the SNr may not be solely caused by the STN hyperactivity. SNc DA neurons extend their dendrites deep into the SNr, where tyrosine-hydroxylase-positive DA dendrites and parvalbumin-positive GABAergic neurons closely intermingle^38–40^. Although there is some disagreement^41^, some reports suggest that dendritically released DA can inhibit the activity of SNr neurons^42,43^. Hence, our SNc DA lesions would release SNr neurons from local DA control and by this means also contribute to the neuronal hyperactivity found in our parkinsonian animals. Our results highlight that the SNr has a central role to play physiologically and in the context of Parkinson’s disease, independently of the influence of the STN, which may have fundamental relevance (see below).

Our network analysis showed that the SNr had short latency nociceptive responses, significantly faster than those measured in the STN and significantly longer than those measured in the PBN. This analysis suggests that the SNr is not only an output structure of the basal ganglia, but also a major input structure, at least regarding nociceptive signals as reported by Yang and colleagues^44^. These signals are likely to be relayed by the PBN, confirming the direct projection described by Schneider and collaborators^17^ between the PBN and the SNr. In the context of action selection, the PBN-SNr pathway and SNr short latency nociceptive responses have major significance. The SNr is part of the brain’s interrupt circuitry, terminating behaviors that achieve a negative outcome, of which pain is a clear example. Activating the SNr may represent a fast way, similar to the activation of an emergency red button, to stop the current behavior. This may allow the selection of a more appropriate action, computed by the basal ganglia network^45,46^, to deal with the source of harmful events. With enhanced nociceptive processing in the SNr in the context of Parkinson’s disease, the interruption of ongoing behavior would be even faster and may thus underlie the lower pain threshold observed in Parkinson’s disease patients.

We showed that the SNr exhibited increased phasic nociceptive responses and/or firing rate and thus an increased output activity after DA lesions. Our experiments did not measure GABA release directly at the level of SNr targets, including the PBN, however with hyperactive SNr GABAergic projection, it is likely that GABA release in target structure will be increased. We thus expected to find a dysfunction at the level of the PBN in our models. Interestingly, we found that PBN activity was impacted in the Total DA lesion group only, in which reduced nociceptive responses were observed. This suggests that the tonically elevated firing rate and the increased nociceptive responses in the SNr, as found in the Total DA lesion group, is a key element impacting PBN nociceptive processing. Our Western Blot results add an additional key element to explain reduced PBN nociceptive processing in the Total DA group. According to our results, SNr hyperactivity may increase the amount of GABA released in the PBN, leading to the increased expression of GABA_A_ receptors, in animals with total DA lesions. This further indicates that the elevated expression of GABA_A_ receptors in the PBN in the Total DA lesion group, is a key element which may underlie the reduction of nociceptive responses and increased nociceptive threshold. It is interesting to note that reduced nociceptive processing in the PBN and the increased latencies to the first sign of discomfort observed in the hot plate test in this group both indicate a general hypoalgesia, while the direct link between both remains to be fully elucidated.

Parkinson’s disease is classically associated with sensory disturbances which have been assessed both clinically and in animal models of Parkinson’s disease. Studies in patients with Parkinson’s disease have reported a diminished ability in the two-point discrimination task^47^, abnormal joint position sense^48^, and altered mechanical stimulation thresholds^48,49^. However, the literature on nociception in the context of Parkinson’s disease is more conflicted. While a large majority of studies report decreased pain thresholds and therefore increased pain sensitivity in Parkinson’s disease patients^7,50,51^, there are reports of increased pain thresholds, hence the possibility of decreased pain sensitivity cannot be ignored^52^. It has to be noted that most clinical studies are performed on Parkinson’s disease patients after a diagnosis based on motor symptoms. Among other neuronal consequences, DA degeneration is therefore at an advanced level at the time of assessment.

Pre-clinical studies also report conflicting results. Similar to studies in Parkinson’s disease patients, a large majority of pre-clinical studies tend to show a hyperalgesia in rodent models of Parkinson’s disease^53–63^. However, our results and those from others show hypoalgesia^64–66^. The main difference between studies showing hyper- versus hypo- algesia is in the models used. Hypoalgesias have been reported in animal models where the DA loss is restricted to the SNc, while hyperalgesias have been mainly observed following a DA degeneration at the level of the ventral tegmental area (VTA) or in models where the toxin was injected within the medial forebrain bundle (MFB) leading to a depletion of the entire ascending DA systems. The MFB model of Parkinson’s disease has been used in a large majority of the experiments assessing nociception in the rat which has contributed to the general conclusion of hyperalgesia. It is important to note that the VTA was preserved in both of our models and the projection to the nucleus accumbens was spared (Supplementary Fig. 1). According to this observation, we thus suggest that the variable results observed in rodent models of Parkinson’s disease could be linked to the DA structures impacted by the lesion. This hypothesis is clinically important as the DA degeneration in Parkinson’s disease is progressive, starting from the lateral part of SNc, then extending over the entire structure, finally reaching the VTA in later stages^67,68^. While it is often suggested that the pain symptoms in Parkinson’s disease may provide an early marker of the disease^69^, they may also represent an additional marker of DA degeneration, with a hypoalgesia indicating a DA loss located in the SNc and a hyperalgesia indicating a DA loss extending beyond this structure, to the mesolimbic pathway. This mechanistic explanation is centered on the involvement of the DA systems in nociception. Additional experiments are however crucially needed to fully evaluate the involvement of the DA systems and other systems in the development of pain symptoms in Parkinson’s disease. Indeed, peripheral alterations such as nociceptor degeneration (Aδ or C fibers)^70^ or loss of epidermal terminal nerves^52,71^ observed in patients with Parkinson’s disease, as well as the degeneration of the noradrenergic or serotonergic systems involved in pain processing^72^, are certainly contributing to the general control of nociceptive thresholds in Parkinson’s disease.

Our results have a second clinical relevance as they indicate a possible mechanism for central neuropathic pain symptoms, based on our characterization of neuro-adaptations in the PBN and findings in the literature, according to the following observations:

- We have measured some of the primary classical cellular markers of neuro-adaptation, and have shown that both DA lesion groups exhibit an increase in the density of filopodial spines and branched spines, the most immature and mature form of spines, respectively. These results suggest an increased turn-over of spine development and an anatomical re-organization in the PBN following a partial and total DA loss in the SNc, potentially to compensate for the inhibitory influence from the SNr and to maintain normal function (indeed, nociceptive responses were unaffected in the Partial DA lesion group). We have also shown that the PSD at the level of the PBN in both DA lesion groups was altered compared to the sham group. Yu and Blas^34^ found that morphological changes in the PSD are associated with an increase in inhibitory communication. The thickness of the PSD, which was decreased in both DA lesion groups, has also been shown to be correlated with synaptic activity^73^. These findings have never been reported in the literature previously and will now require additional experiments to fully characterize the molecular and anatomical changes within the PBN in the context of Parkinson’s disease and understand better their impact on the PBN function, including finer analysis specifically of the SNr-PBN pathway in relation to the spino-PBN pathway.
- Inhibition of the SNr had no impact on tonic or phasic PBN neuronal activity in the Sham group, questioning the role of this pathway under normal physiological conditions. However, in both DA lesion groups, a similar pharmacological inhibition of the SNr induced a substantial firing rate and nociceptive response rebound in the PBN due to the previously described neuro-adaptations. We also found that cross-correlated activity between the SNr and PBN strongly increased in both DA lesion groups (30 and 49%) compared to the Sham group (12%) during the control period. Both sensory rebound and cross-correlated activity indicate that the functional link between the SNr and the PBN becomes pathologically stronger in the context of Parkinson’s disease.
- At a clinical level, Terao and collaborators^74^ hypothesized that SNr hyperactivity in Parkinson’s disease patients may be ‘leaky’, oscillating between active and release phases during the day.

Similar to our previous hypothesis concerning hyperactivity in the superior colliculus in patients with Parkinson’s disease^75^, we thus suggest that transient release of SNr inhibitory control over the PBN would be comparable to the SNr pharmacological blockade in our study. If similar neuro-adaptative mechanisms develop within the PBN in patients with Parkinson’s disease, SNr release suggested by Terao and collaborators^74^ could then generate a similar sensory rebound within the PBN and generate the phantom pain sensations described in central pain in Parkinson’s disease. Furthermore, the sensory rebound in nociceptive processing in the PBN following SNr inhibition implies that the function of projection from the SNr to the PBN has been altered by the DA lesions. In terms of basal ganglia function, SNr output conveys the decision about the selected action to the thalamus and sensori-motor structures of the brainstem, with reduced SNr activity disinhibiting channels at the thalamic and brainstem levels that have been selected for expression whilst increased SNr activity inhibits those that have not^45,46^. According to our results, when SNr activity decreases to enable selection, in those instances where the PBN is one of the targets, the decrease in SNr firing is likely to generate an abnormal response in the PBN (cf. the difference between the effects of intra-nigral muscimol in the Sham group and the DA lesion groups). In the case of the PBN, this may lead to abnormal levels of pain associated with whatever action has been selected. Further experiments are now needed to evaluate the anatomical and functional state of the PBN in Parkinson’s disease patients.

We feel it is important to briefly draw attention to a couple of limitations in the present work we report. Firstly, the electrophysiological experiments were performed on the right side of the brain, the side of the 6-OHDA lesion. This bias was due to a methodological constraint at the level of the electrophysiological set up. The implantation of multiple electrodes required a combination of multiple skull entry points and approach angles, forcing us to focus on a single side. A note of caution should then be taken in extrapolating our results to the left side of the brain as evidence for pain-related lateralization has been reported, especially regarding the left and right amygdala. However, pain processing shows a clear lateralization toward the right hemisphere, which is the focus of our experiments^76–78^. A second note of caution is required since we used only male rats, and thus care should be taken in extrapolating our results to females, in which nociceptive responses have been reported to be quantitatively different (i.e. they have a lower nociceptive threshold)^79,80^.

We focused our Western Blot analysis on GABA receptors according to the GABAergic nature of the projection between the SNr and the PBN. However, our Golgi-cox analysis revealed structural changes at the level of the dendritic spines involving possible additional neuroplasticity impacting the glutamatergic system. While our PSD analysis revealed that most of the PSD analyzed were located on the shaft of dendrites, therefore suggesting their GABAergic nature, our result cannot guarantee the PSD analyzed are from synapses innervated by afferents from the SNr. Finer analysis is now needed combining tract tracing technique and electron microscopy to clearly identify SNr and non SNr synapses. Neuroplasticity outside the basal ganglia, to our knowledge, had hardly been studied and our work provide the first evidence of such mechanism in the PBN in the context of Parkinson’s disease. Further work is now required to fully characterize the functional, anatomical and molecular impact of a DA lesion on activity in the PBN in relation to the SNr, as well as in other structures connected to the basal ganglia.

Finally, although we have focused our discussion of the functional changes observed in the PBN in the total dopaminergic lesion group on the pathological link between the SNr and the PBN and increased expression of GABA_A_ receptors, we cannot exclude the possibility that other factors contribute to the changes we report in parabrachial function. For example, altered nociceptive integration in the spinal cord as shown in the MFB rat model of Parkinson’s disease^62^ may also contribute to the changes. Similarly, the PBN is strongly interconnected with the amygdala and structures in the brainstem such as the superior colliculus which are known to be impacted in Parkinson’s disease and rat models of the disease^26,75,81^. Dysfunction of this afferent network could also impact the activity of the PBN although the extent to which this contributes to the changes we see has to be fully elucidated.

In conclusion, we have found that a primary nociceptive structure from the brainstem, the PBN, shows anatomical and functional alterations following a DA lesion in the SNc. We suggest these alterations are due to hyperactivity of the SNr which is caused, at least in part, by hyperactivity of the STN, demonstrated in the present experiments. Studying each element of a nociceptive network linking the PBN, the STN and the SNr, in the context of Parkinson’s disease, revealed that the SNr appears to have a more central role than the STN in the development of the dysfunctional re-organisation in the PBN, a re-organisation which we suggest is part of the mechanism underlying central neuropathic pain in Parkinson’s disease. The pathological influence of the SNr after a partial DA lesion is further accentuated by STN hyperactivity after a total DA lesion, causing a functional nociceptive impairment. These results also highlight the lack of known detail concerning the precise anatomical and functional link between the SNr and the PBN, or between the SNr and its other direct targets such as sensori-motor structures from the brainstem, despite their significance in the context of Parkinson’s disease.

## Methods

### Animals

Male Long Evans rats (*N* = 74) were used for these experiments. They were kept in the same standard environmental conditions (12 h light/dark cycle) at a constant temperature of 22 °C, with food and water provided ad libitum. The experiments were carried out in accordance with the policy of Lyon 1 University, the Grenoble Institute des Neurosciences (GIN) and French legislation. Experiments were conducted in compliance with the European Community Council Directive of November 24, 1986 (86/609/EEC). The research was authorized by the Direction Départementale des Services Vétérinaires de l’Isère - Ministère de l’Agriculture et de la Pêche, France (Coizet, Véronique, PhD, permit number 381003). Every effort was made to minimize the number of animals used and their suffering during the experimental procedures. All procedures were reviewed and validated by the “Comité éthique du GIN n°004” as agreed by the research ministry.

### 6-OHDA lesions

Male Long Evans rats (*n* = 74) were anaesthetized with isoflurane (5% for the induction and 1–2% for the surgery) and placed in a stereotaxic frame. Their heads were shaved, cleaned and disinfected with Vétédine® and a midline incision was performed on the scalp to allow access to the skull. A burr-hole was then drilled above the substantia nigra pars compacta of the right hemisphere, and 6-hydroxydopamine hydrobromide (Sigma-Aldrich, 2.6 mg.mL^−1^) injections were made with a 30 G cannula connected with polyethylene tubing to a 10 µL Hamilton syringe, driven by an infusion pump at 0.5 µL/min. At the end of the injection, the cannula was left in place for 5 min to allow diffusion of the toxin. Animals were divided in three groups according to the injection protocol: (i) A group with a partial dopaminergic lesion (Partial DA lesion, *n* = 24) that received 1.0 µL of 6-OHDA at the following coordinates: Anterior-posterior (AP): +3,0 mm; Medio-lateral (ML): +2.4 mm and Dorso-ventral (DV): +2.4 mm from interaural zero; (ii) A group with a total dopaminergic lesion (Total DA lesion, *n* = 21) that received 3.0 µL of 6-OHDA at the following coordinates: AP: +3.0 mm; ML: +2.1 mm and DV: +2.5 mm from interaural zero; (iii) A control group that received the vehicle (sterile 0.9% NaCl) instead of the 6-OHDA (Sham, *n* = 29) at the following coordinates: AP: +3.0 mm; ML: +2.4 mm and DV: +2.4 mm from interaural zero according to the rat brain atlas of Paxinos and Watson^82^.

### Electrophysiology

After a delay of at least 3 weeks to allow the 6-OHDA-induced lesion to stabilize, rats (*N* = 70; Sham = 25; Partial DA lesion = 22; Total DA lesion = 23) were anaesthetized with an intra-peritoneal injection of ethyl carbamate (urethane, 1.25 g/kg, Sigma-Aldrich) and placed in a stereotaxic frame. Body temperature was maintained at 37 °C with a thermostatic heating blanket throughout the recording sessions. Two stainless steel electrodes (E363-1, Plastics One, Roanoke, VA) were inserted into the left hindpaw for noxious stimulation. One was placed under the skin of the plantar surface of the foot, the other was placed under the skin of the medial aspect of the lower leg. Three craniotomies were then performed to allow simultaneous access to the STN, SNr and PBN. Extracellular voltage excursions were amplified, band-pass filtered (300 Hz–10 kHz), digitized at 10 kHz and recorded directly onto computer disc using a Micro 1401 data acquisition system (Cambridge Electronic Design [CED] Systems, Cambridge, UK) running CED data capture software (Spike 2).

#### STN single unit-recordings

Extracellular single-unit recordings were obtained from STN neurons (simultaneously with multi-unit recordings from the SNr and single unit recordings from the PBN), contralateral to the stimulated hindpaw, using glass micropipettes pulled via a vertical electrode puller (Narishige Laboratory Instruments Ltd. Tokyo, Japan) with a tip diameter <1.0 µm and impedances ranging from 5–20MΩ (measured at 135 Hz in 0.9% NaCl). Electrodes were filled with 0.5 M saline and 2% Pontamine Sky Blue (BDH Chemicals Ltd., Poole, UK). The electrode was lowered into the brain at the following coordinates (AP): 3.6–4.16 mm caudal to Bregma; (ML): 2.0–3.0 mm lateral to midline and (DV): 6.8–8.2 mm ventral to the brain surface according to the rat brain atlas^82^. The STN electrode was lowered until a putative STN neuron was identified on the basis of several criteria as previously described (Pautrat et al.)^16^.

#### SNr multi-unit recordings

Extracellular multi-unit recordings were obtained from the SNr (simultaneously with single unit recordings from the PBN and STN), contralateral to the stimulated hindpaw, using a tungsten-Parylene electrode (Phymep, Paris, France) coupled to a 30 G stainless cannula filled with the GABA_A_ agonist muscimol (0.25 µg/µL). An angled trajectory was used - the electrode was tilted medially by 28°, entering the brain via the left hemisphere to end in the right hemisphere SNr at the following coordinates: (AP): 5.4 mm caudal to Bregma; (ML): 1.8 mm lateral to midline. The SNr was encountered at 8.4 mm ventral to the brain surface according to the rat brain atlas^82^. Muscimol microinjections of 0.5 µL were made via a 10 µl Hamilton syringe driven by an infusion pump at 0.5 µL/min.

#### PBN single unit-recordings

Extracellular single-unit recordings were obtained from the PBN (simultaneously with multi-unit recordings from the SNr and single unit recordings from the STN), contralateral to the stimulated hindpaw, using a glass micropipette pulled via a vertical electrode puller (Narishige Laboratory Instruments Ltd. Tokyo, Japan) with a tip diameter <1.0 µm and impedances ranging from 5–20MΩ (measured at 135 Hz in 0.9% NaCl). Electrodes were filled with 0.5 M saline and 2% Pontamine Sky Blue (BDH Chemicals Ltd., Poole, UK). The electrode was lowered into the brain using an angled approach with the electrode tilted at 30° towards the rostral aspect of the brain. The electrode entered the brain at the following coordinates (AP): 11.0–12.0 mm caudal to Bregma; (ML): 2.0–3.0 mm lateral to midline. PBN neurons were encountered 5.0–5.6 mm below the brain surface according to the rat brain atlas^82^.

#### Noxious stimulation

Electrical stimulation was performed using the hindpaw contralateral to the 6-OHDA-induced lesion and recording sites. Electrical stimulation consisted of 5 mA pules (2 ms duration) presented 120 times at 0.5 Hz. The nociceptive nature of this protocol was ascertained in previous studies^16,26,28^. The activity of the cells (single-unit in STN/PBN and multi-unit in the SNr) was recorded during a control period (120 trials of sham stimulation) and then during the application of noxious footshocks (120 trials). For the muscimol experiments, an injection of muscimol was made in the SNr. Typically, a change in local SNr multi-unit activity was seen within 2 min of the injection. Noxious electrical footshocks were applied throughout this period, until either the effects of the drug wore off, or the PBN cell was lost. After a complete trial, further PBN neurons were tested in the same way.

#### Electrophysiological analysis

Following the recording sessions, the artefact of the stimulation was removed from our recordings using a script developed by Cambridge electronic design limited (CED, script ArtRem, https://ced.co.uk/fr/downloads/scriptsigedit), peri-stimulus time interval histograms (PSTHs) were constructed based on SNr multi-unit (bin width 1 ms) and STN/PBN single-unit data (bin width 10 ms). PSTHs were imported into an Excel program (Peter Furness, University of Sheffield^16,18,24,28,29^) which allowed us to analyze the following response characteristics: (i) firing rate: the mean number of single/multi-unit events during the 500 ms prior to the footshock; (ii) Response latency: the latency of a nociceptive evoked response marked as the point when the value of post-stimulation events exceeded 1.96 S.D. (single-unit) or 3.00 S.D. (multi-unit) of the pre-shock firing rate; (iii) Response duration: the time between response onset (latency) and response offset marked as the point when post-stimulation activity returned to a value below 1.96/3.0S.D. of the pre-shock firing rate; (iv) Peak amplitude of the response: the maximum amplitude during the response; (v) Response magnitude: the mean number of single/multi-unit events between response onset and offset. In other words, the area under the curve during the response.

#### Histology

The final recording site of the STN and PBN electrodes (glass micropipettes) was marked with the iontophoretic injection of Pontamine Sky Blue, performed by passing a constant cathodal current of 27.5 µA through the electrode for 20 min. The position of the tungsten electrode was marked with a small lesion made by passing a constant anodal current of 10 µA DC current through the electrode for 1 min. Animals were then euthanized by overdose of pentobarbital (60 mg/kg, i.p.) and perfused with 250 mL of 0.9% saline followed by 250 mL of 4% formaldehyde. Brains were dissected and postfixed in the same fixative overnight at 4 °C before being cryo-protected in 20% sucrose solution for 36 h. Brains were then snap-frozen in −50 °C isopentane and stored at −20 °C. Two series of 30 µm coronal sections were taken. One was directly mounted onto gelatin-coated slides and stained with cresyl-violet. The other was placed in anti-freeze solution and stored at −20 °C for immunohistochemistry. Recording sites were then checked under the microscope and recording trajectories were reconstructed onto sections of the atlas of Paxinos and Watson^82^.

### Firing patterns analysis

STN/PBN firing rate before and after the noxious stimulation was assessed during the 500 ms before the sham (control period) and noxious stimulations (stimulation period). The data were imported into MATLAB, binned and compared using a Wilcoxon signed-rank test. STN/PBN firing patterns were also assessed using MATLAB according to the methodology developed by Piallat et al.^83^ Neurons were classified as irregular, regular or bursting according to the interspike distributions and autocorrelograms. Burst activity showed a wide or bimodal interspike interval distribution and a significant single peak in the autocorrelation function. Irregular activity was characterized by a wide interspike interval distribution and a flat autocorrelogram. Regular activity was characterized by a narrow interval interspike distribution and an autocorrelogram with multiple regular peaks.

### Cross correlogram analysis

Because cross-correlogram histograms are not normally distributed, their statistical significance was assessed non-parametrically using surrogate distributions of firing patterns obtained from actual recordings. For each pair of recordings of several minutes, surrogate firing patterns were created by resampling randomly in time the action potentials (single-units or multi-units) according to a standard uniform distribution. This procedure broke any time dependency between both regions, but conserved their mean firing activity. Then surrogate cross-correlogram histograms were computed from the surrogate firing patterns on between −1.3 and 0.5 s (see Supplementary Fig. 5). This procedure was repeated 99 times and the maximum value of the surrogate cross-correlograms for each time bin was conserved, in order to assess the significance threshold of having a correlated response at *p*-value 0.01. Recorded and surrogate cross-correlograms were then visually reviewed and evidence for cross-correlated activity was decided when the recorded cross-correlogram was above the significance threshold in the time range [−50 50] ms for at least 10 ms.

### Hot-plate test

Nociceptive thermal threshold assessments were performed using the hot-plate test in lesioned and control animals (Sham = 23; Partial DA lesion = 20; Total DA lesion = 18). The test consists of placing the rat on a metal pan warmed to 53 °C. The latency to lick the hind-paw(s), jump, vocalize or stomp was measured. Additionally, the rat’s general behavior was assessed during this test and exploratory behavior checked in order to evaluate the presence of motor deficits. Parameters such as rearing, full turns and change of direction when turning were counted.

### 6-OHDA lesions analysis

The extent of the DA denervation induced by 6-OHDA injections in the SNc was determined using tyrosine hydroxylase (TH) immunohistochemistry. To reveal TH, the sections were washed and incubated in a blocking solution containing 0.1 M phosphate buffer (PB) with 0.3% triton X-100 (TX), 2.5% Bovine Serum Albumin (BSA) and 5% normal horse serum (NHS) for 2 h before being transferred overnight to a solution of 0.1 M PB-TX 0.3% with 1% BSA and 2% NHS containing primary mouse monoclonal TH antibody, diluted 1:3,000 (Chemicon, Hampshire, UK). The following day, sections were washed in 0.1 M PB and incubated with the secondary antibody, biotinylated antimouse made in horse (at a dilution of 1:1,000 in 0.1 M PB-TX 0.3% with 2% NHS) for 2 h. Following further washes in 0.1 M PB, the sections were exposed to the elite Vectastain ABC reagent (Vector Laboratories, Burlingame, CA, USA) diluted 1:100 in PB-TX 0.3%, for 2 h. Again following washes in 0.1 M PB, immunoreactivity was revealed by exposure to nickel enhanced 3,3'-Diaminobenzidine (DAB, Sigma-Aldrich) for 2 min, which produced a black reaction product. Sections were then mounted onto gelatin-coated slides, dehydrated through a graded ethanol series and cleared in xylene before being coverslipped with DPX. TH-immunolabelling of DA neurons and terminals was evaluated using a light microscope (Nikon, Eclipse 80i, TRIBVN, Chatillon, France) coupled to the ICS Framework computerized image analysis system (TRIBVN, 2.9.2 version, Chatillon, France). For quantification, TH-labeled coronal sections of the SNc (AP −5.3 mm to −5.8 mm from Bregma) and striatum (AP 0.20 mm to −0.30 mm from Bregma) were digitized using a Pike F-421C camera (ALLIED Vision Technologies Stradtroda, Germany). Optical densities were then measured for the denervated hemisphere (right) and non-denervated hemisphere (left) of operated animals. Optical densities of the lesioned hemisphere were then compared to the non-lesioned hemisphere in each animal.

### Golgi-cox staining

Rats (Sham = 5; Partial DA lesion = 4; Total DA lesion = 3) were euthanized with pentobarbital (60 mg/kg, i.p.) and perfused as previously described. Brains were collected and placed in 4% formaldehyde overnight for post-fixation, after which they were placed in Golgi-Cox solution (1% potassium dichromate, 1% potassium chromate, 1% mercuric chloride) for 2 days. The solution was then replaced by a fresh one and brains were immersed for a further 12 days. The incubation (total of 14 days) took place in the dark at room temperature. Brains were then washed in 1% potassium dichromate for 24 h, placed in 20% sucrose for 24 h for cryo-protection and then snap-frozen in −50 °C isopentane. Two sets of slices were taken. The first consisted of 30 µm sections centered on the striatum and SNc (AP: +0.2/−0.3 mm; AP: −5.2/−5.8 mm after Bregma, respectively), collected in anti-freeze solution and stored and −20 °C for TH immunohistochemistry. The second set of slices consisted of 80 µm sections centered on the PBN (AP: −8.8 mm to −10.0 mm after Bregma) transferred directly onto gelatin-coated slides. The greater thickness was optimal for preservation of the tissue and observation of spines on the secondary and tertiary dendritic segments. The slides were then developed with Kodak rapid-fix diluted at 1:6 in distilled water, dehydrated in alcohols and coverslipped in DPX. Sections were allowed to dry at room temperature for 72 h and were then cleaned and scanned.

Slides stained with the Golgi-Cox technique were scanned using Axio Scan Z at 400x magnification. 10 Z-stacks with a 2.0 µm step were taken, centered on the PBN region. Images were imported into Reconstruct software that utilizes the distinct geometric characteristics of spines as the basis for their categorization (see Risher et al. for details)^32^. Dendritic sections of neurons clearly located in the PBN were analyzed. Parameters such as spine length, width and number were measured. The software then classified the spine morphology automatically based on these measurements, displaying information such as dendrite length/width, total spine count, percentage of mushroom/ stubby/ filopodia/ branched spines, and spine density. A minimum of 5 dendrites located on different neurons were measured for each animal. In total, the length of analyzed dendritic sections was ~600 µm/animal.

### Electron microscopy

Three rats per group (total *N* = 9) were examined using electron microscopy. Sections centered on the PBN were first washed with PB, post-fixed with 1% osmium tetroxide in PB, 0.1 M pH 7.2, for 25 min, stained in 1% uranyl acetate at pH 4 for 40 min in the dark and dehydrated through a graded ethanol series before being embedded in epoxy resin (Flukka) between two sheets of Aclar film. Samples were left to polymerize for 3 days at 60 °C. Ultrathin sections of the sample (50 nm) were cut with an ultra-microtome (Leica Ultracut). Sections were then stained in uranyl acetate and lead citrate before being viewed under a transmission electron microscope at 80 kV (JEOL 1200EX). Images of synapses were taken at 12, 000 x and 50, 000 x magnification with a digital camera (Veleta, Olympus) (*n* = 50 / animal) and morphometric parameters were measured with iTEM software (Olympus, Soft Imaging System). Experiments were performed in collaboration with the Electron Microscopy Facility of the Grenoble Institute des Neurosciences (MEC).

### Western Blot

#### Tissue collection

Rats were anesthetized with isoflurane (5% for the induction and 1–2% for the surgery). The brain was excised, rapidly frozen using Isopentane (−80 °C) and stored at −80 °C. Thick sections (80 µm) centered on the PBN were taken using a Leica CM 1850 cryostat. Both lateral and medial parts of the PBN were then isolated using a tissue punch and all samples were stored at −80 °C until further use.

#### Preparation of samples

PBN samples were homogenized in cold 10% sodium dodecyl sulfate solution and a protease inhibitor cocktail (Sigma-Aldrich) using a Sonicator with a 1 s on/off cycle for 15 rounds. Samples were then centrifuged at 15,000 × g for 15 min at 4 °C. The clear supernatant was collected, and the protein concentration was determined using a BCA protein Assay Kit (Thermofisher, USA) with bovine serum albumin (BSA) as the standard.

#### Western Blot analysis

Expression of GABA_A_ receptors was determined using Western Blot analysis. Protein samples were mixed with 4X sample loading buffer (Laemmeli with beta mercaptoethanol), boiled at 95 °C for 10 min, cooled and then centrifuged at 10,000 × g for 2 min. Equal amounts of protein (20 μg) for each group were resolved using 10% sodium dodecyl sulfate-polyacrylamide gel electrophoresis (SDS-PAGE). Proteins were then electrophoretically transferred to a polyvinylidene fluoride membrane (PVDF) (GE healthcare, Germany) for 50 min. The transferred proteins were then blocked with 3% BSA in TBS-T (7.4 pH) containing Tris-HCl (50 mM), NaCl (150 mM), Tween-20 (0.1%) for 2 h at room temperature. The blot was then incubated overnight at 4 °C with the following primary antibodies: rabbit anti-GABAAR α1 (1∶2000, Sigma-Aldrich, Germany), and rabbit anti-GAPDH (1:2000, Sigma-Aldrich, USA). After incubation, the blots were washed with TBS-T three times for 10 min each. The blots were incubated with anti-rabbit horseradish peroxidase-conjugated secondary antibody (1:10,000, Cell Signaling, USA) for 2 h. After washing three times (each wash for 10 min) ECL Plus substrate (Thermofisher, USA) was applied to the blots for 5 min in the dark. The blots were revealed using Chemidoc Bio-Rad and the protein expression intensity was assessed by integrated optical density (IOD). To account for possible differences in total protein load, the results of the measurements were presented as a ratio of IOD of GABA_A_ receptor protein to the IOD of the respective GAPDH using ImageJ software (Bio-rad Laboratories).

### Statistics

All statistic tests were performed using GraphPad Prism v6.01 software. The Shapiro-Wilk test was used to assess the normality of the different data distributions. The statistical reliability of differences between the three groups for all data was assessed using either an ANOVA analysis followed by Tukey’s post-hoc multiple comparisons or a Kruskal-Wallis analysis followed by Dunn’s post-hoc multiple comparisons according to the normality of the distribution of the data. Comparisons of response durations, response latencies, peak amplitudes and response magnitudes before and after injections of muscimol in the SNr were tested using a two-way-ANOVA on repeated measures followed by Bonferroni’s post-hoc multiple comparison test. Chi-square (*χ*²) tests were used to assess if the data distribution of the firing patterns, nociceptive response types and correlated activities were different between our three animal groups. Results are presented as: mean ± SEM. Significance was set at *P* < 0.05, two-tailed.

### Reporting summary

Further information on research design is available in the Nature Research Reporting Summary linked to this article.

## Supplementary information

Supplementary Material

Reporting Summary2

## Data Availability

The data that support the findings of this study are available from the corresponding author upon request.

## Code availability

Matlab scripts used to analyse the data are freely available on the ImaGIN platform website (https://f-tract.eu/software/imagin/; copy of the source code archived at https://github.com/elifesciences-publications/ImaGIN2_source_code). Matlab scripts use to analyse the cross-correlation data is available from the corresponding author upon request.
