# Supplementary material for: Altered parabrachial nucleus nociceptive processing may underlie central pain in Parkinson’s disease
Source: NPJ Parkinsons Dis. 2023 May 26;9:78. doi: 10.1038/s41531-023-00516-x (PMC10220088; doi:10.1038/s41531-023-00516-x)
Supplement: Supplementary file 1 — Supplementary Material [file 41531_2023_516_MOESM1_ESM.pdf]

Supplementary Materials:

A – Supplementary results (Page 1 to 5).

B – Supplementary figures (Page 6 to 10).

C – Supplementary tables (Page 11 to 14).

A – Supplementary results

I. Tonic nociceptive processing in the STN

The noxious stimulations did not change the baseline firing rate of STN neurons in the three experimental groups (2-way ANOVA on repeated measures, $F[1,156] = 1.98$, followed by Bonferroni's multiple comparison, $p= 0.17$ in Sham, $p=0.67$ in Partial DA lesion, $p=0.36$ in total DA lesion). However, we previously demonstrated that the baseline firing rate changed differently at a cell level in control rats, with cells increasing, decreasing or not changing their firing rate with the introduction of the nociceptive foot-shock (UP, DOWN, NO CHANGE cells, Pautrat et al., 2018). We therefore performed individual statistical analysis to compare the change of the baseline firing rate before and after the introduction of the nociceptive footshock (Wilcoxon test) and confirmed the presence of those three different groups of cells. The proportion of the cells in the three experimental groups can be found in the Supplementary Table 3.

This processing was not disrupted by the dopaminergic lesions as this distribution was not statistically different between the groups ($\chi2$: 7.873, $p = 0.096$).

II. STN response types

STN neurons showed complex phasic responses to noxious stimulations delivered in the contralateral hindpaw (120 occurrences at 0,5Hz). STN phasic responses to the footshock presented the same pattern of response as previously described (Pautrat et al., 2018):

1. Monophasic excitation (N = 106) which could be subdivided into three categories:

- -Monophasic short-latency (mean ± SEM: 43 ± 4.6 ms), short-duration excitation (mean ± SEM: 31.1 ± 1.8 ms) (n = 49),

- Monophasic short-latency (mean ± SEM: 33 ± 4 ms), long duration excitation (mean ± SEM: 176 ± 20 ms) (n = 43),

- Monophasic long-latency (mean ± SEM: 200 ± 5.8 ms), long duration excitation (mean ± SEM: 200 ± 44.7 ms) (n = 14).

2. Biphasic response (n = 33) composed of two successive short latencies (mean ± SEM: 22.6 ± 4.5 ms) excitations (n = 32) or an excitation followed by an inhibition (n = 1).

3. Tri-phasic response (n = 20) characterized by an initial short-latency (mean ± SEM: 20 ± 4.5 ms), short duration excitation, then an inhibition or a marked reduction in firing rate followed by a third late-latency and long lasting excitation (+/-/+)(total duration mean ± SEM: 160 ± 25.5 ms).

III. SNr tonic nociceptive processing

The introduction of the noxious stimulation significantly increased SNr baseline firing rate in the sham (mean ± SEM, no stimulation = 56.84 ± 3.03 Hz – stimulation = 67.01 ± 3.99 Hz;), partial DA lesion (mean ± SEM, no stimulation = 63.24 ± 6.25 Hz – stimulation = 76.61 ± 7.77 Hz) and total DA lesion group (mean ± SEM, no stimulation = 74.93 ± 5.17 Hz – stimulation =

87.62 ± 5.64 Hz) ((Figure 4B) (2-way ANOVA on repeated measures, $F[1,279] = 31.82$, followed by Bonferroni's multiple comparison, $p<0.01$).

IV. SNr phasic nociceptive response types.

The noxious stimulation induced short latencies phasic responses that were characterized as follow:

1. Monophasic responses (n = 119) which could be subdivided into two categories:

- Monophasic short-latency (mean ± SEM: 14 ± 0.001 ms), short-duration excitation (mean ± SEM: 21 ± 0.001 ms) responses (n = 110) (figure 4Ca).
- Monophasic short-latency (mean ± SEM: 18 ± 0.003 ms), long-duration (mean ± SEM: 121 ± 0.31 ms) responses (n = 9) (not illustrated).

2. Biphasic responses with short latencies (n = 113, mean ± SEM: 13 ± 0.0005 ms) composed of two successive excitations (n = 105, Figure 4Cb) or an excitation followed by an inhibition (n = 8) (not illustrated).

3. Tri-phasic responses (n = 52) characterized by:

- Tri-phasic +/+/+: three successive excitations (n = 42) (latency: mean ± SEM: 12 ± 0.003 ms) (Figure 4Cc).
- Tri-phasic +/-/+: an initial short-latency (mean ± SEM: 18 ± 0.001 ms), short-duration excitation, then an inhibition or a marked reduction in firing rate followed by a third late-latency and long lasting excitation (n = 10) (figure 4Cd).

4. The previous phasic responses were sometimes followed by late excitations (n = 24) and inhibition (n = 1). Those phases had latencies ranging from 85 to 801 ms (mean ± SEM: 241.16 ± 42.10 ms) and durations ranging from 85 to 745 ms (mean ± SEM: 301 ± 37.40 ms).

V. PBN firing patterns

The PBN cells firing patterns were in majority of irregular and bursting patterns (Figure 5B). The proportion of the firing patterns according to the three experimental groups can be found in the Supplementary Table 4.

The remaining cells in the three experimental groups were of regular or a mixture of the other patterns. Unlike the STN, the progressive decreased and increased proportions of irregular and bursting patterns with the level of DA denervation were not statistically significant ($\chi 2$: 4.113 $p = 0.128$).

VI. PBN phasic nociceptive response types.

Following the noxious stimulations, PBN neurons recorded in this experiment showed various and complex phasic responses with several patterns of response which could be categorized into:

1. Monophasic short latencies excitation (mean ± SEM: 18.69 ± 0.17 ms) with either a short (n = 88, mean ± SEM: 28.4 ± 0.004 ms) (Figure 6a) or long duration (n = 9, mean ± SEM: 263.4 ± 0.07 ms) (not illustrated).

2. Biphasic responses with an initial short latency excitation (mean ± SEM: 14.31 ± 0.001 ms) followed by a second excitation (n = 39) (Figure 6b) or inhibition (n = 19) (Figure 6c).

3. Several complex short latencies (mean ± SEM: 18.2 ± 0.002 ms) triphasic responses:

- Triphasic +/-/+ (n = 17) characterized by an initial short duration excitation, then an inhibition or a marked reduction in firing rate followed by a third late-latency and long lasting excitation (Figure 6d),

- Triphasic +/+/+ (n = 12) composed of three successful excitations (Figure 6e).

- Triphasic +/+/- (n = 7) with two successful excitations followed by a long lasting inhibition or a marked reduction in firing rate (Figure 6f).

4. We recorded 3 cells with a phasic inhibition following the nociceptive footshock and the previous phasic responses were sometimes followed by late excitations (n = 13) or inhibitions (n = 2). Those phases had latencies ranging from 114 to 815 ms (mean ± SEM: 408.16 ± 56.75 ms) and durations ranging from 25 to 480 ms (mean ± SEM: 243.06 ± 35.14 ms).

Unlike the STN and SNr, PBN phasic responses types to the footshock were not differently distributed among the three experimental groups ($\chi 2$: 6.52 p = 0.163).

B – Supplementary figures

Supplementary figure 1

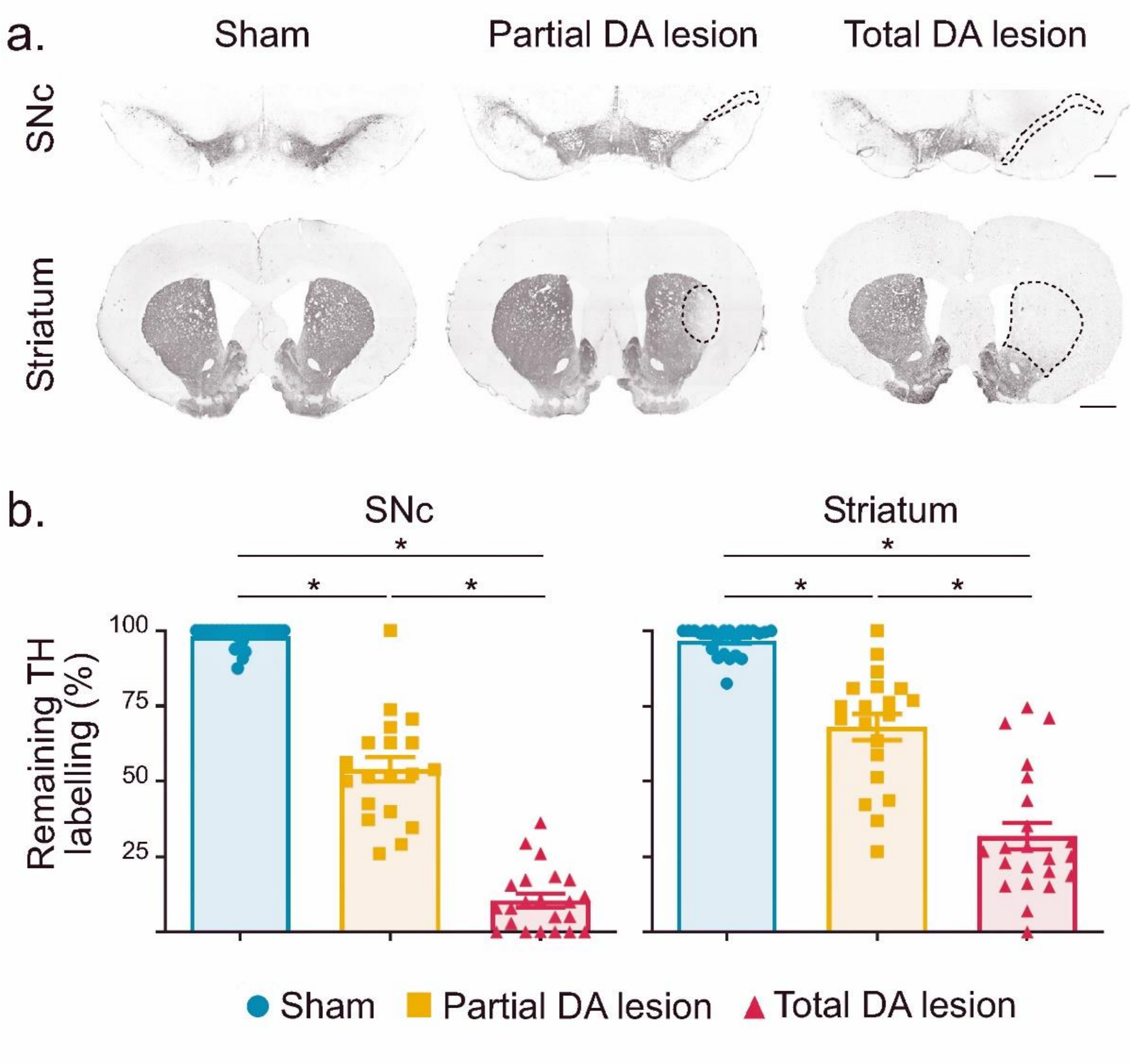

Supplementary Figure 1: Histological analysis of the 6-OHDA lesions. Individual examples of coronal sections immunostained against TH in the SNc and the striatum (A) illustrating the difference in TH labelling between the three experimental groups. The dotted lines in the SNc and the striatum highlight the DA denervation. (B) Histograms of the mean percentage (± SEM) of remaining TH labeling in the SNc (*left*) and the striatum (*right*) for Sham (*n* = 29, blue), Partial (*n* = 24, orange) and Total (*n* = 21, red) DA lesion groups. Scale bars = 1 mm.

Abbreviations: DA: dopamine; SNc: substantia nigra pars compacta.

Supplementary figure 2

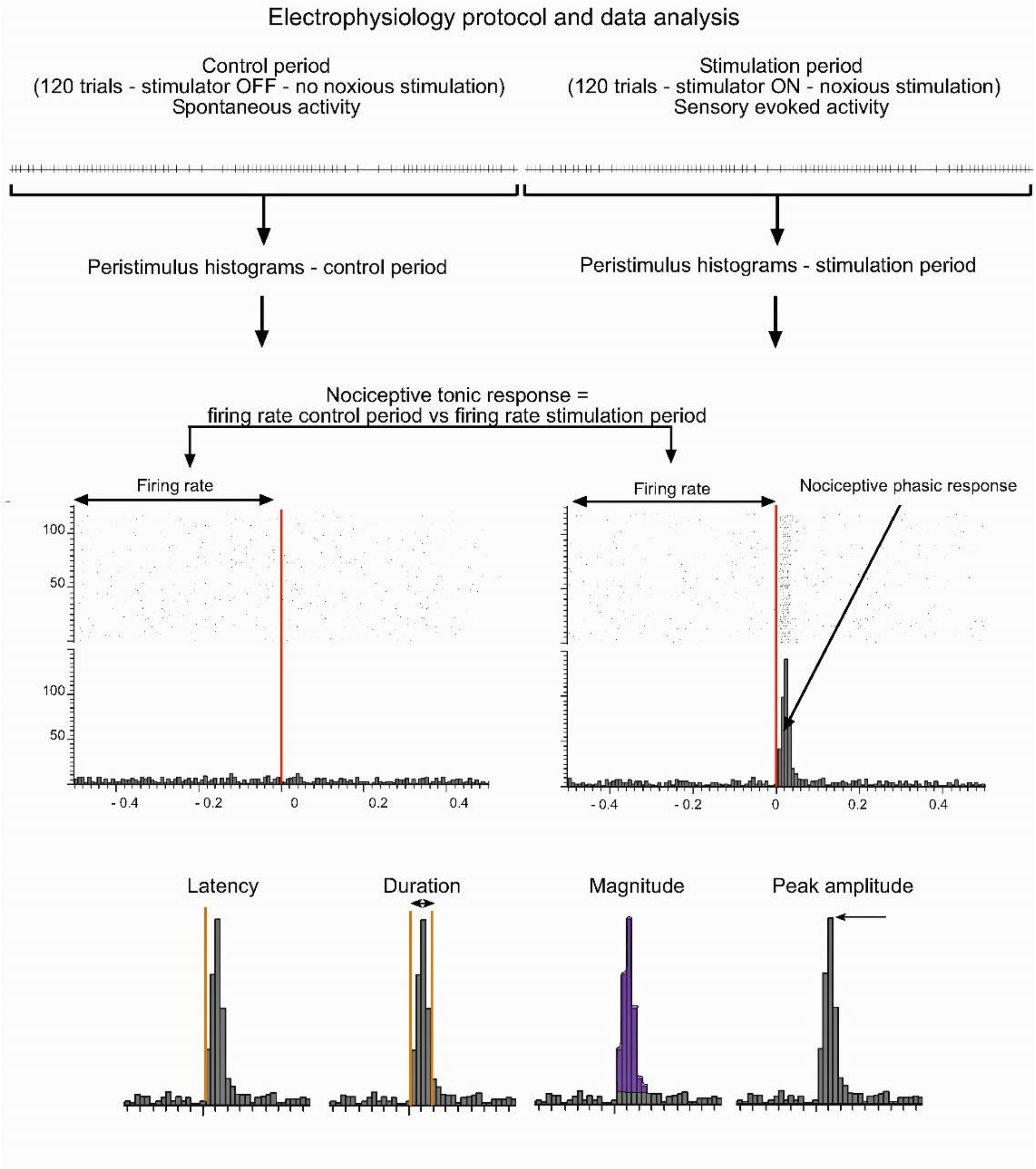

Supplementary Figure 2: Schematic of the electrophysiological protocol and data analysis. The recordings were conducted during a control period to evaluate the spontaneous neuronal activity of the recorded cells and during a stimulation period during which a noxious footshock was delivered at an intensity of 5.0 mA (0.5 Hz, 2 ms, 120 stimulations). Peristimulus histograms were then constructed for both periods for data analysis including the firing rate of the cells, measured during the 500 ms prior to the sham or noxious stimulations (indicated by the red lines), and the nociceptive phasic response, i.e. the temporary change of neuronal activity following the noxious stimulation. From this phasic response, the latency, duration, magnitude and maximum amplitude were measured. A change in tonic nociceptive processing corresponds to a change of firing rate between the control and the stimulation period.

Supplementary figure 3

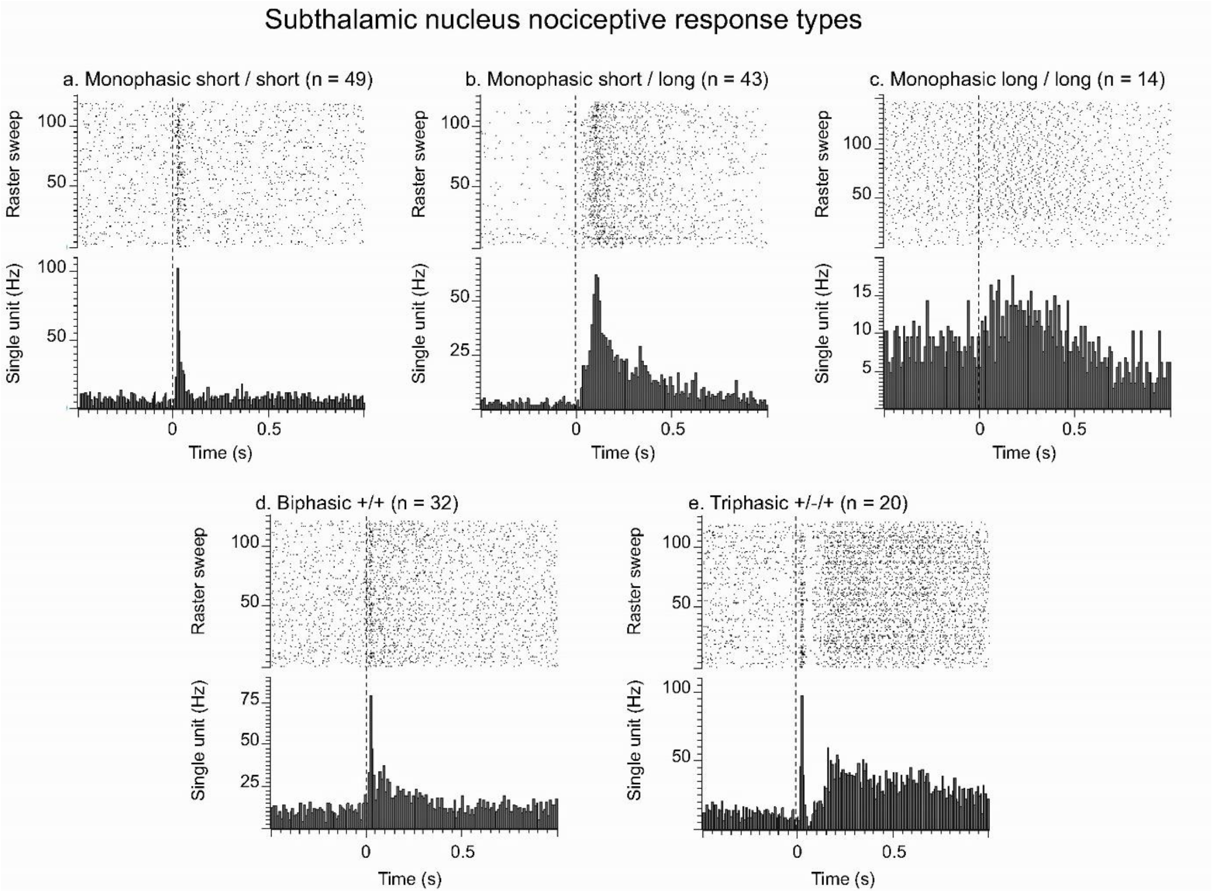

Supplementary Figure 3: Subthalamic nucleus phasic responses. Peristimulus histograms showing individual cases of monophasic short latency / short duration (a), monophasic short latency / long duration (b), monophasic long latency / long duration (c), biphasic +/+ (d) and triphasic +/-/+ (e), phasic noxious stimulus-evoked responses in the STN. The dashed vertical line indicates the onset time of the noxious footshock. The *n* associated with each histogram indicates the number of cases exhibiting that class of response.

Supplementary figure 4

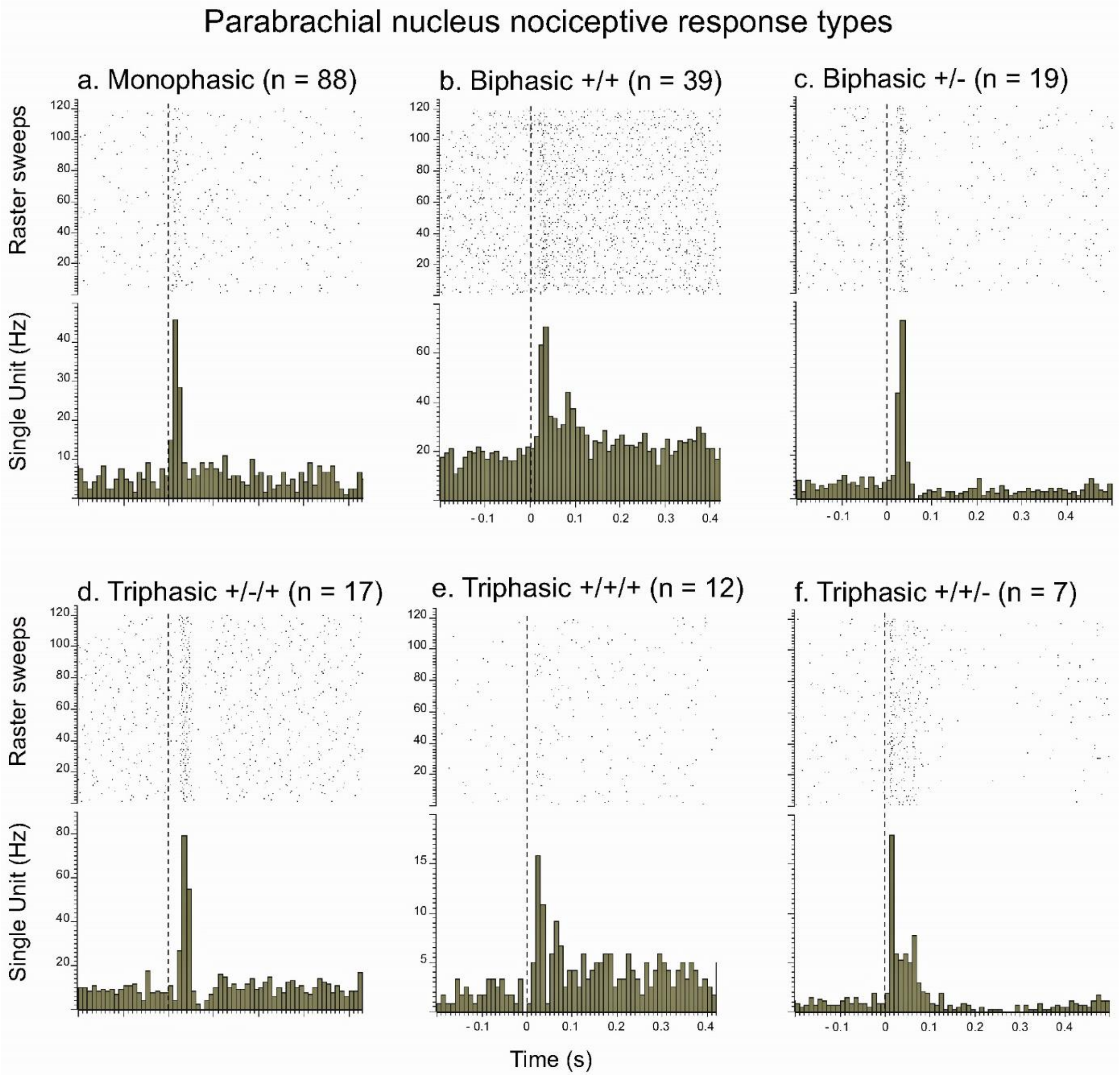

Supplementary Figure 4: Parabrachial nucleus phasic responses. Peristimulus histograms showing individual cases of monophasic short latency/short duration (a), biphasic +/+ (b), biphasic +/- (c), triphasic +/-/+ (d), triphasic p+/+/+ (e) and triphasic +/+/- (f) phasic noxious stimulus-evoked responses in the PBN. The dashed vertical line indicates the onset time of the noxious footshock. The *n* associated with each histogram indicates the number of cases exhibiting that class of response.

Supplementary figure 5

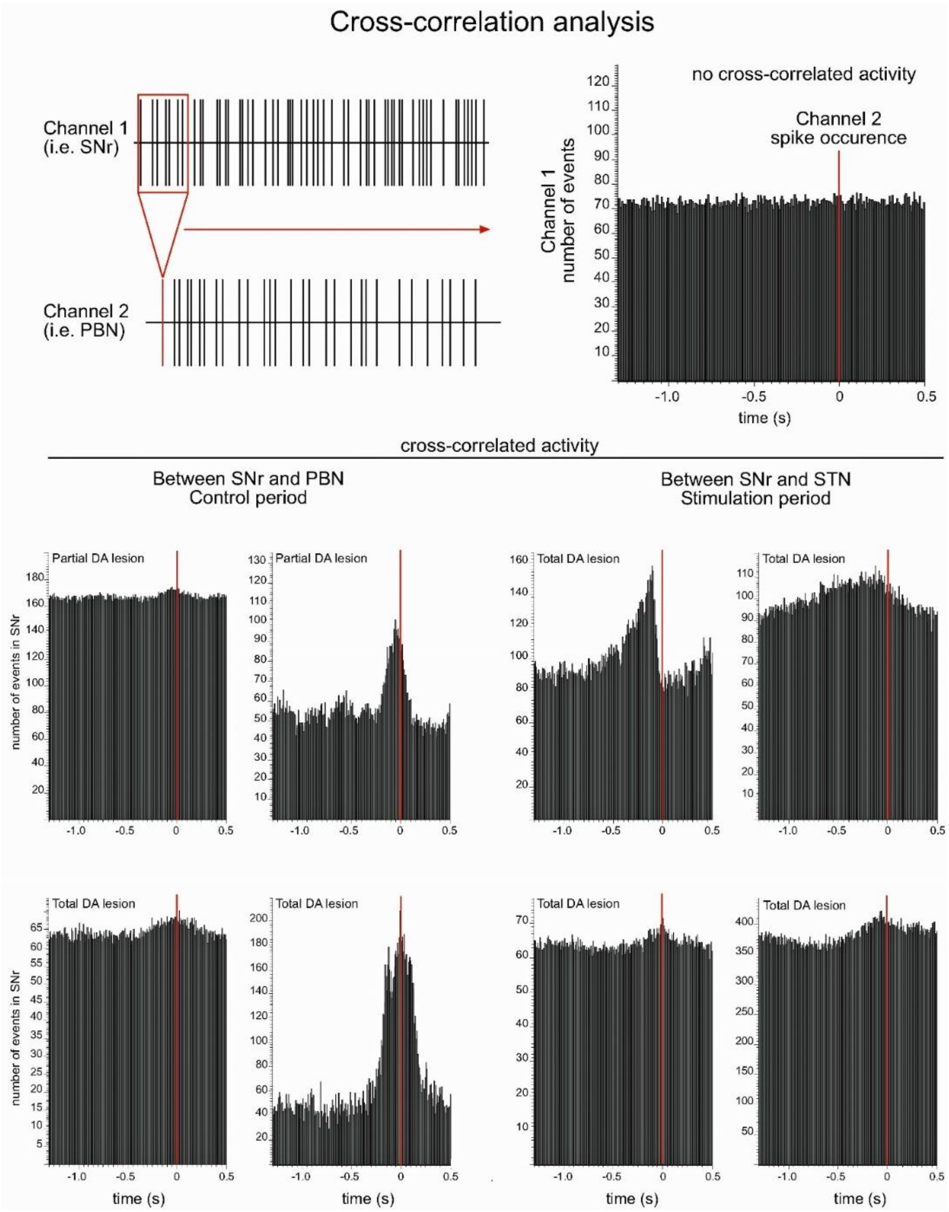

Supplementary Figure 5: Cross-correlation histograms. Top panel: Illustration of the cross-correlation analysis (Left). The occurrence of a single unit spike on channel 2 (i.e. PBN) was used to trigger the plotting of spikes on channel 1 (i.e. SNr). The cross-event histogram is the average (in Hz) of channel 1 events before and after the occurrence of spikes from channel 2. If the spiking of two recorded neurons are independent, the cross-event histograms would be expected to be flat without an asymmetry or a central peak (Right panel). Bottom panel: Illustration of cross-event histograms in which a significant peak or asymmetry was found during the control period between the SNr and the PBN (Left panels) (PBN spike occurrence: red line) and during the stimulation period between the SNr and the STN (STN spike occurrence: red line) in both DA lesion groups.

C – Supplementary tables

Supplementary Table 1

	Groups	Number of recordings	Latency (ms)	Duration (ms)	Baseline FR (Hz) – Control period	Baseline FR (Hz) – Stimulation period	Magnitude (Hz)	Peak Amplitude (Hz)
STN	Sham	61	33.28 ± 4.97	127.10 ± 16.47	7.20 ± 0.67	8.64 ± 1.10	7.48 ± 0.63	29.10 ± 3.89
	Partial DA lesion	52	30.69 ± 5.04	151.80 ± 27.68	8.10 ± 0.91	8.71 ± 1.55	10.23 ± 1.24	40.56 ± 6.75
	Total DA lesion	46	43.04 ± 6.79	139.70 ± 18.12	11.48 ± 1.24*	11.85 ± 1.34$^{\#*}$	10.01 ± 0.92	38.92 ± 5.02
SNr	Sham	124	13.11 ± 0.43	40.97 ± 3.67	56.84 ± 3.03	66.62 ± 4.01	88.72 ± 5.62	286.90 ± 13.18
	Partial DA lesion	66	12.64 ± 0.63	39.92 ± 8.94	63.60 ± 6.29	77.65 ± 7.92	133.90 ± 10.92*	404.00 ± 28.58*
	Total DA lesion	94	14.68 ± 0.49 $^{\#}$	33.52 ± 3.86	74.93 ± 5.17$^{\#*}$	87.62 ± 5.65*	115.10 ± 9.86*	356.96 ± 21.75*
PBN	Sham	72	11.97 ± 1.15	116.70 ± 19.23	7.81 ± 0.91	8.01 ± 0.85	19.19 ± 2.53	58.33 ± 7.87
	Partial DA lesion	73	16.53 ± 1.05*	107.00 ± 17.04	7.71 ± 0.96	8.03 ± 0.85	17.53 ± 1.94	44.59 ± 4.18
	Total DA lesion	49	26.64 ± 3.15*	116.20 ± 20.93	8.32 ± 1.28	8.34 ± 1.20	8.67 ± 0.83$^{\#*}$	27.55 ± 2.65$^{\#*}$

Supplementary Table 1: Firing rate and phasic response parameters (mean ± SEM) in the STN, SNr and PBN.

Supplementary Table 2

STN-PBN	control period					stimulation period				
	yes	no	N	yes (%)	no (%)	yes	no	N	yes (%)	no (%)
sham	**4**	**44**	**48**	8	92	**20**	**28**	**48**	42	58
partiel	**5**	**22**	**27**	19	81	**5**	**21**	**26**	19	81
total	**1**	**20**	**21**	5	95	**0**	**21**	**21**	0	100

STN-SNr	control period					stimulation period				
	yes	no	N	yes (%)	no (%)	yes	no	N	yes (%)	no (%)
sham	**14**	**19**	**33**	42	58	**23**	**10**	**33**	70	30
partiel	**9**	**11**	**20**	45	55	**11**	**9**	**20**	55	45
total	**8**	**26**	**34**	24	76	**27**	**7**	**34**	79	21

PBN-SNr	control period					stimulation period				
	yes	no	N	yes (%)	no (%)	oui	non	N	yes (%)	no (%)
sham	**4**	**29**	**33**	12	88	**19**	**14**	**33**	58	42
partiel	**19**	**44**	**63**	30	70	**42**	**21**	**63**	67	33
total	**22**	**23**	**45**	49	51	**27**	**18**	**45**	60	40

Supplementary Table 2: Percentage of correlated and un-correlated activity between each pair of structure for the three experimental groups during the control and stimulation period.

Supplementary Table 3.

Groups	UP cells	Down cells	No change cells
Sham	42 % (26/61)	7 % (4/61)	51 % (31/61)
Partial DA lesion	46 % (24/52)	21 % (11/52)	33 % (17/52)
Total DA lesion	46% (21/46)	9 % (4/46)	46 % (21/46)

Supplementary Table 3: Proportion of the up, down and no change cells in the three experimental groups.

Supplementary Table 4.

Groups	Firing patterns	
	Irregular	Burst
Sham	58 % (42/72)	24 % (17/72)
Partial DA lesion	49 % (36/73)	26 % (19/73)
Total DA lesion	41 % (20/49)	39 % (19/49)

Supplementary Table 4: Proportion of the irregular and burst firing patterns according to the three experimental groups.
