# Supplementary material for: Altered parabrachial nucleus nociceptive processing may underlie central pain in Parkinson’s disease
Source: NPJ Parkinsons Dis. 2023 May 26;9:78. doi: 10.1038/s41531-023-00516-x (PMC10220088; doi:10.1038/s41531-023-00516-x)
Supplement: Supplementary file 2 — Reporting Summary2 [file 41531_2023_516_MOESM2_ESM.pdf]

nature portfolio

Corresponding author(s): COIZET Véronique

Last updated by author(s): Apr 16, 2023

Reporting Summary

Nature Portfolio wishes to improve the reproducibility of the work that we publish. This form provides structure for consistency and transparency in reporting. For further information on Nature Portfolio policies, see our Editorial Policies and the Editorial Policy Checklist.

Statistics

For all statistical analyses, confirm that the following items are present in the figure legend, table legend, main text, or Methods section.

n/a	Confirmed	
☐	☒	The exact sample size (n) for each experimental group/condition, given as a discrete number and unit of measurement
☐	☒	A statement on whether measurements were taken from distinct samples or whether the same sample was measured repeatedly
☐	☒	The statistical test(s) used AND whether they are one- or two-sided<br>*Only common tests should be described solely by name; describe more complex techniques in the Methods section.*
☐	☒	A description of all covariates tested
☐	☒	A description of any assumptions or corrections, such as tests of normality and adjustment for multiple comparisons
☐	☒	A full description of the statistical parameters including central tendency (e.g. means) or other basic estimates (e.g. regression coefficient) AND variation (e.g. standard deviation) or associated estimates of uncertainty (e.g. confidence intervals)
☒	☐	For null hypothesis testing, the test statistic (e.g. F, t, r) with confidence intervals, effect sizes, degrees of freedom and P value noted<br>*Give P values as exact values whenever suitable.*
☒	☐	For Bayesian analysis, information on the choice of priors and Markov chain Monte Carlo settings
☒	☐	For hierarchical and complex designs, identification of the appropriate level for tests and full reporting of outcomes
☒	☐	Estimates of effect sizes (e.g. Cohen's d, Pearson's r), indicating how they were calculated

Our web collection on statistics for biologists contains articles on many of the points above.

Software and code

Policy information about availability of computer code

Data collection	Electrophysiology: Spike 2.
Data analysis	Electrophysiology: Spike 2.<br>Golgi: Reconstruct software<br>Western blot: ImageJ software<br>Prisme

For manuscripts utilizing custom algorithms or software that are central to the research but not yet described in published literature, software must be made available to editors and reviewers. We strongly encourage code deposition in a community repository (e.g. GitHub). See the Nature Portfolio guidelines for submitting code & software for further information.

Data

Policy information about availability of data

All manuscripts must include a data availability statement. This statement should provide the following information, where applicable:

- Accession codes, unique identifiers, or web links for publicly available datasets
- A description of any restrictions on data availability
- For clinical datasets or third party data, please ensure that the statement adheres to our policy

The data that support the findings of this study are available from the corresponding author upon request. Matlab scripts used to analyse the data are freely available on the ImaGIN platform website (https://f-tract.eu/software/imagin/ ; copy of the source code archived at https://github.com/elifesciences-publications/ImaGIN2_source_code). Matlab scripts use to analyse the cross-correlation data is available from the corresponding author upon request.

Research involving human participants, their data, or biological material

Policy information about studies with human participants or human data. See also policy information about sex, gender (identity/presentation), and sexual orientation and race, ethnicity and racism.

Reporting on sex and gender	n/a
Reporting on race, ethnicity, or other socially relevant groupings	*Please specify the socially constructed or socially relevant categorization variable(s) used in your manuscript and explain why they were used. Please note that such variables should not be used as proxies for other socially constructed/relevant variables (for example, race/ethnicity should not be used as a proxy for socioeconomic status).*<br>*Provide clear definitions of the relevant terms used, how they were provided (by the participants/respondents, the researchers, or third parties), and the method(s) used to classify people into the different categories (e.g. self-report, census or administrative data, social media data, etc.)*<br>*Please provide details about how you controlled for confounding variables in your analyses.*
Population characteristics	n/a
Recruitment	n/a
Ethics oversight	n/a

Note that full information on the approval of the study protocol must also be provided in the manuscript.

Field-specific reporting

Please select the one below that is the best fit for your research. If you are not sure, read the appropriate sections before making your selection.

☒ Life sciences ☐ Behavioural & social sciences ☐ Ecological, evolutionary & environmental sciences

For a reference copy of the document with all sections, see nature.com/documents/nr-reporting-summary-flat.pdf

Life sciences study design

All studies must disclose on these points even when the disclosure is negative.

Sample size	Sample size was determined according to our previous experience in behavioral and electrpohysiological assessment.
Data exclusions	The absence of a 6-OHDA lesion despite the injections of the toxin was the only exclusion criteria.
Replication	We have not tested replication.
Randomization	The animals have been randomly tested in all the procedure.
Blinding	Investigators were blinded when testing the animals.

Reporting for specific materials, systems and methods

We require information from authors about some types of materials, experimental systems and methods used in many studies. Here, indicate whether each material, system or method listed is relevant to your study. If you are not sure if a list item applies to your research, read the appropriate section before selecting a response.

Materials & experimental systems

n/a	Involved in the study
☐	☒ Antibodies
☒	☐ Eukaryotic cell lines
☒	☐ Palaeontology and archaeology
☐	☒ Animals and other organisms
☒	☐ Clinical data
☒	☐ Dual use research of concern
☒	☐ Plants

Methods

n/a	Involved in the study
☒	☐ ChIP-seq
☒	☐ Flow cytometry
☒	☐ MRI-based neuroimaging

Antibodies

Antibodies used	Western blot: rabbit anti-GABAAR α1 (1：2000, Sigma-Aldrich, Germany), and rabbit anti-GAPDH (1:2000, Sigma-Aldrich, USA). TH antibody (Chemicon, Hampshire, UK).
Validation	These anti-body have been used and validated scientifically in our lab for a long time.

Animals and other research organisms

Policy information about studies involving animals; ARRIVE guidelines recommended for reporting animal research, and Sex and Gender in Research

Laboratory animals	Rats - Long Evans - 3 month old
Wild animals	n/a
Reporting on sex	we used only male rats, and thus care should be taken in extrapolating our results to females, in which nociceptive responses have been reported to be quantitatively different (i.e. they have a lower nociceptive threshold).
Field-collected samples	n/a
Ethics oversight	The experiments were carried out in accordance with the policy of Lyon 1 University, the Grenoble Institute des Neurosciences (GIN) and French legislation. Experiments were conducted in compliance with the European Community Council Directive of November 24, 1986 (86/609/EEC). The research was authorized by the Direction Départementale des Services Vétérinaires de l'Isère - Ministère de l'Agriculture et de la Pêche, France (Coizet, Véronique, PhD, permit number 381003). Every effort was made to minimize the number of animals used and their suffering during the experimental procedures. All procedures were reviewed and validated by the "Comité éthique du GIN n°004" as agreed by the research ministry.

Note that full information on the approval of the study protocol must also be provided in the manuscript.
